# Scale Effects between Body Size and Limb Design in Quadrupedal Mammals

**DOI:** 10.1371/journal.pone.0078392

**Published:** 2013-11-08

**Authors:** Brandon M. Kilbourne, Louwrens C. Hoffman

**Affiliations:** 1 Committee on Evolutionary Biology, University of Chicago, Chicago, Illinois, United States of America; 2 Department of Geology, Field Museum of Natural History, Chicago, Illinois, United States of America; 3 Department of Animal Sciences, Stellenbosch University, Stellenbosch, Western Cape Province, South Africa; Monash University, Australia

## Abstract

Recently the metabolic cost of swinging the limbs has been found to be much greater than previously thought, raising the possibility that limb rotational inertia influences the energetics of locomotion. Larger mammals have a lower mass-specific cost of transport than smaller mammals. The scaling of the mass-specific cost of transport is partly explained by decreasing stride frequency with increasing body size; however, it is unknown if limb rotational inertia also influences the mass-specific cost of transport. Limb length and inertial properties – limb mass, center of mass (COM) position, moment of inertia, radius of gyration, and natural frequency – were measured in 44 species of terrestrial mammals, spanning eight taxonomic orders. Limb length increases disproportionately with body mass via positive allometry (length ∝ body mass^0.40^); the positive allometry of limb length may help explain the scaling of the metabolic cost of transport. When scaled against body mass, forelimb inertial properties, apart from mass, scale with positive allometry. Fore- and hindlimb mass scale according to geometric similarity (limb mass ∝ body mass^1.0^), as do the remaining hindlimb inertial properties. The positive allometry of limb length is largely the result of absolute differences in limb inertial properties between mammalian subgroups. Though likely detrimental to locomotor costs in large mammals, scale effects in limb inertial properties appear to be concomitant with scale effects in sensorimotor control and locomotor ability in terrestrial mammals. Across mammals, the forelimb's potential for angular acceleration scales according to geometric similarity, whereas the hindlimb's potential for angular acceleration scales with positive allometry.

## Introduction

**Figure 1:**
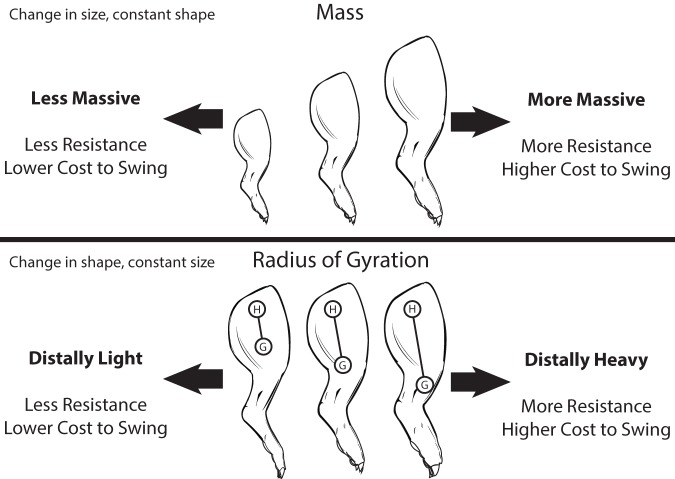
The separate influence of limb size and shape on the cost of swinging the limb. With increasing limb mass, the relevant measure of size, the cost of swinging the limb increases. With a longer radius of gyration, represented by the distance between points H and G, the cost of swinging the limb increases. The radius of gyration is the relevant measure of limb shape (i.e., mass distribution) for a swinging limb. Limb mass (m) and radius of gyration (r) determine the limb's moment of inertia (MOI), or its quantified resistance to swinging, through the following function: MOI  =  mr^2^.

Across terrestrial mammals lies an astounding diversity of limbs, in terms of the relative proportions of segments, the position of muscle origins and insertions, posture, and function. These aspects of limb morphological diversity, along with body size, determine the overall size and shape of whole limbs, and notably limb shape and size determine the limb's intrinsic resistance to being swung back and forth (Fig. 1). Mass, one measure of limb size, is the limb's resistance to linear acceleration (i.e., translational or straight line movements). The limb's center of mass is a measure of its mass distribution – a measure of shape – along its proximo-distal length. Regarding rotational or swinging movements, a limb's resistance to angular acceleration is its moment of inertia (MOI). With respect to MOI, the radius of gyration is an alternative measure of the limb's proximo-distal mass distribution. Notably, the radius of gyration (*r*) is a function of the ratio of limb MOI to mass (*m*):
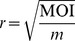
(1)


An increase in mass or MOI or a distal shift in the limb's mass distribution shifts results in an increased cost of swinging the limb, as the limb muscles must exert greater force to accelerate and decelerate the limb. Limb mass, MOI, and COM position in concert determine the limb's natural frequency, its optimal frequency of oscillation at which gravitational potential and kinetic energy are maximally exchanged, and the point where muscular effort to swing the limb is minimized [1], [2].

**Figure 2:**
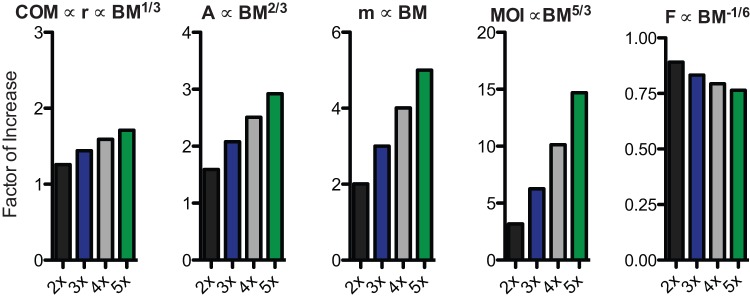
Predictions of how limb inertial properties scale against body mass under geometrically similar scaling. Among the inertial properties, mass is denoted by ‘m’, radius of gyration by ‘r’, and natural frequency by ‘F’. Muscle cross-sectional area is denoted by ‘A’ and body mass by ‘BM’. Note y-axes not to same scale.

Though the metabolic cost of swinging the limbs has only become clear in recent years, being between 8 to 33% of total metabolic locomotor costs [3]–[5], functional morphologists and biomechanists have had a longstanding interest in how whole limb size and shape influence terrestrial locomotor costs and limb movements [2], [6]–[12]. However, previous studies have never rigorously examined the influence of body size on whole limb inertial properties. Body size dependent increases in limb inertial properties have a grave possibility to limit the locomotor ability of larger bodied mammals, especially under isometric scaling. Under isometric scaling, muscle cross-sectional area is proportional to (body mass)^2/3^ [13]; however, limb mass and MOI are proportional to (body mass)^1.0^ and (body mass)^5/3^, respectively (Fig. 2). Thus with increasing body mass, the limb's inertia – or resistance to acceleration – increases at a much greater rate than the force potential of muscles. In light of this, geometric similarity of limb inertial properties would leave larger bodied mammals with a decreased capability to accelerate and decelerate their limbs and, as a possible consequence, with an overall diminished locomotor ability (e.g,, maximum attainable speed, maneuverability, or agility).

However, previous studies have found that the mass-specific locomotor costs of larger mammals are diminished as compared to small mammals. The mass-specific cost of transport (COT), the amount of metabolic energy consumed to transport one unit body mass over one unit distance, decreases with increasing body size in terrestrial mammals [14]–[16]. Differences in stride frequency between small and large mammals appear to be critical to the lower mass-specific COT of large mammals. Across small and large mammals, the amount of mass-specific metabolic energy consumed per stride is constant [17]–[18]. However, small mammals take more strides per unit distance, which results in relatively greater values of mass-specific COT in smaller mammals [16], [17]–[19]. In addition to scale effects in stride frequency, scale effects in limb inertial properties may also underlie the increased locomotor economy (in terms of mass-specific metabolic costs) of larger-bodied mammals. Given the oscillatory nature of limb movements, negative allometry of limb inertial properties would reduce the muscle-supplied forced necessary to accelerate and decelerate relative to body mass and could contribute to the low mass-specific COT of larger bodied mammals. Moreover, the link between stride frequency and the scaling of metabolic locomotor costs adds further plausibility to the notion that limb rotational inertia may be related to locomotor costs.

However, the mass-specific cost of transport may also be strongly influenced by by kinetic energy lost in foot-ground collisions and the need for muscles to replace this lost energy [20]–[22]. During locomotion, as a quadruped's whole body COM makes contact with the ground through its limb, it loses kinetic energy. In order to maintain constant velocity, this lost kinetic energy must be replaced, either by elastic storage and recovery or by the active generation of mechanical energy by muscles (if not by both of these means). Thus to lower metabolic locomotor costs, animals must minimize energy lost during the collisions of their limbs with the ground. This minimization of energy loss is achieved by minimizing differences in the orientation of traveling velocity (i.e., velocity vector orientation) prior to and after the limb's contact with the ground, and by using multiple minimal loss collisions (i.e., footfalls) as opposed to fewer collisions with greater energy losses (see Ruina et al. [20]; Bertram & Gutman [21] for a much more detailed explanation of this model). Note though, that this model only concerns the stance phase of locomotion and does not concern swing phase or its associated metabolic costs [20].

To assess how limb size and shape co-vary with changes in body size, we will assess scale effects in whole limb inertial properties. Using a sample of species that vary widely in body size and ecological specialization, we will determine general scaling trends for quadrupedal mammals. To determine if the scaling of limb inertial properties deviate from isometry, we will test scaling relationships against geometric similarity, a null model dictating that proportions remain constant with changes in size. By comparing scaling relationships to this null model, we will identify whether limb inertial properties have the potential to influence the scaling of the COT and infer how scale effects in inertial properties otherwise influence differences in locomotor ability between small and large mammals.

## Materials and Methods

### Sampling and carcass condition

**Figure 3:**
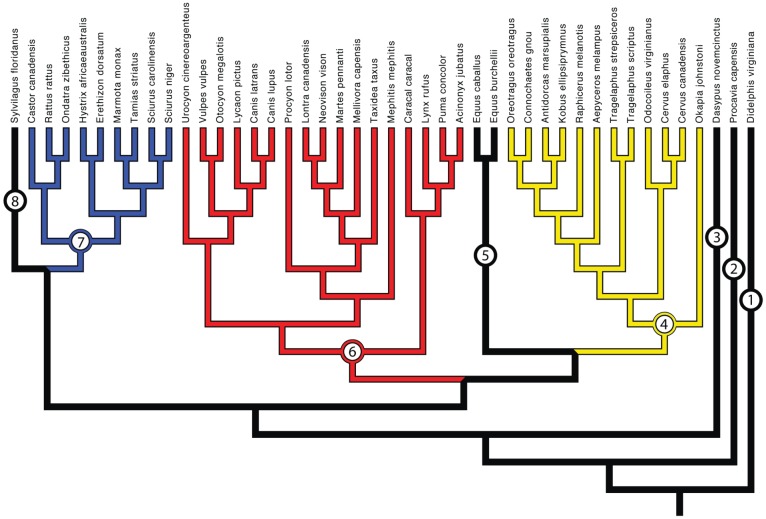
Phylogeny of sampled mammalian species. The major lineages, or taxonomic orders, sampled include Didelphimorphia (1), Hyracoidea (2), Cingulata (3), Artiodactyla (4), Perissodactyla (5), Carnivora (6), Rodentia (7), and Lagomorpha (8). Mammalian orders separately analyzed are highlighted in red, blue, and yellow. Tree topology primarily based upon Meredith et al. [45]. However, the topologies of specific mammalian orders were taken from other published studies (see File S1 for more details).

Carcasses of adult individuals of 44 mammalian species were obtained from the Field Museum of Natural History, the Iziko South African Museum, the University of Stellenbosch, federal, state and local wildlife agencies, and veterinary colleges (Fig. 3 & Table 1). Carcasses were also graciously donated by game farms in South Africa. Specimens were solely studied via collections visits or specimen donation, and no specimens were killed for the express purpose of this study. Dissections were performed within the guidelines for animal tissue research of the University of Chicago's Institutional Animal Care and Use Committee (IACUC). Our specific dissection methodology was approved by the University of Chicago's IACUC (Animal Care & Use Protocol No. 71872). The condition and quality of studied specimens is provided as supporting information in File S1.

**Table 1:** Taxa included in this study, along with sample size, body mass, limb specializations, posture, and source locality.

Common name	Species	N	Body Mass	Specialization	Locality
**Didelphimorphia**					
Virginia opossum	*Didelphis virginiana*	3	3240	Scansorial	USA (Illinois)
**Carnivora**					
Coyote	*Canis latrans*	2	11488	Cursorial	USA (Illinois)
Gray wolf	*Canis lupus*	7	30773	Cursorial	USA (Minnesota)
African hunting dog	*Lycaon pictus*	1	22050†	Cursorial	USA (Illinois)*
Bat-eared fox	*Otocyon megalotis*	1	3000	Cursorial	South Africa (Western Cape)
Gray fox	*Urocyon cinereoargenteus*	4	3745	Cursorial	USA (Illinois, Minnesota)
Red fox	*Vulpes vulpes*	1	3587	Cursorial	USA (Minnesota)
Cheetah	*Acinonyx jubatus*	1	50000†	Cursorial	USA (Illinois)*
Caracal	*Caracal caracal*	3	7050		South Africa (Western Cape)
Bobcat	*Lynx rufus*	4	8960	Cursorial	USA (Illinois, Minnesota)
Mountain lion	*Puma concolor*	1	68039	Cursorial	USA (Illinois)
Striped skunk	*Mephitis mephitis*	1	2386	Generalist	USA (Illinois)
N. American river otter	*Lontra canadensis*	5	7287	Natatorial	USA (Minnesota)
Fisher	*Martes pennanti*	5	4662	Scansorial	USA (Minnesota, Wisconsin)
Ratel	*Mellivora capensis*	2	15100	Fossorial	South Africa (Western Cape)
N. American badger	*Taxidea taxus*	4	6173	Fossorial	USA (Wisconsin)
Raccoon	*Procyon lotor*	5	6179	Scansorial	USA (Illinois, Wisconsin)
**Artiodactyla**					
Impala	*Aepyceros melampus*	2	51750	Cursorial	South Africa (Eastern Cape, Limpopo)
Springbok	*Antidorcas marsupialis*	3	39050†	Cursorial	South Africa (Western Cape)
Black wildebeest	*Connochaetes gnou*	1	107450	Cursorial	South Africa (Eastern Cape)
Waterbuck	*Kobus ellipsiprymnus*	2	238000	Cursorial	South Africa (Limpopo)
Klipspringer	*Oreotragus oreotragus*	2	10500	Cursorial	South Africa (Limpopo)
Grysbok	*Raphicerus melanotis*	1	9750	Cursorial	South Africa (Western Cape)
Bushbuck	*Tragelaphus scriptus*	1	54000	Cursorial	South Africa (Limpopo)
Greater kudu	*Tragelaphus strepsiceros*	2	236500	Cursorial	South Africa (Limpopo)
Elk	*Cervus canadensis*	5	241500	Cursorial	USA (Colorado)
Red deer	*Cervus elaphus*	1	166563†		South Africa (Western Cape)
White-tailed deer	*Odocoileus virginianus*	4	69318	Cursorial	USA (Illinois)
Okapi	*Okapia johnstoni*	1	230001†	Cursorial	USA (Illinois)*
**Cingulata**					
Nine-banded armadillo	*Dasypus novemcinctus*	2	4904	Fossorial	USA (Louisiana)
**Hyracoidea**					
Rock hyrax	Procavia capensis	1	2250	Scansorial	South Africa (Limpopo)
**Lagomorpha**					
Eastern cottontail	*Sylvilagus floridanus*	3	1131	Cursorial/Saltatorial	USA (Illinois)
**Perissodactyla**					
Domestic horse	*Equus caballus*	4	446250	Cursorial	USA (Michigan)
Burchell's zebra	*Equus quagga burchellii*	2	266675		South Africa (Limpopo)
**Rodentia**					
N. American beaver	*Castor canadensis*	5	16810	Natatorial	USA (Illinois, Wisconsin)
N. American porcupine	*Erethizon dorsatum*	1	5540	Scansorial	USA (Wisconsin)
Cape porcupine	*Hystrix africaeaustralis*	2	14936†	Fossorial	South Africa (Western Cape)
Muskrat	*Ondatra zibethicus*	4	1061	Natatorial	USA (Illinois)
Black rat	*Rattus rattus*	4	331	Generalist	USA (Illinois)
Woodchuck	*Marmota monax*	2	3542	Fossorial	USA (Wisconsin)
Gray squirrel	*Sciurus carolinensis*	4	528	Scansorial	USA (Illinois)
Fox squirrel	*Sciurus niger*	2	666	Scansorial	USA (Illinois)
Eastern chipmunk	*Tamias striatus*	4	97	Generalist	USA (Illinois, Wisconsin)

For the purposes of this study, cursorial denotes limb adaptations and behavior regarding high speed or sustained periods of terrestrial locomotion. Scansorial denotes limb adaptations and behavior regarding climbing, whereas natatorial describes limb adaptations and behavior regarding aquatic locomotion. Fossorial indicates limb adaptations and behavior regarding digging, either in search of food items or burrowing. As body mass can greatly vary with geographic location, the source locality for each species is listed with state/province in parentheses. Body mass values listed in grams.

† Body mass taken from Smith et al. [24] and not directly from specimen(s).

* Zoo specimens.

To attain a morphologically diverse sample and identify scaling trends for terrestrial mammals as a whole, we collected data on mammals representing the following locomotor types: generalist, cursorial, scansorial, fossorial, and natatorial. Cursorial indicates taxa specialized for running at high speeds or for prolonged periods, and scansorial denotes taxa specialized for climbing. Fossorial denotes taxa with limbs specialized for digging, whereas natatorial denotes taxa with limbs specialized for swimming. By sampling a range of locomotor types, our aim is to identify for each inertial property whether a single scaling trend governs the scaling of limb inertial properties in terrestrial mammals. Our ultimate goal is to determine what changes occur in limb shape and size alongside changes in body size in terrestrial mammals.

As the scaling patterns are known to vary between different groups within Mammalia [23], we also examined the scaling of limb inertial properties within both taxonomic and functional groups within Mammalia. Taxonomic subgroups analyzed include Carnivora, Artiodactyla, and Rodentia. Cursorial mammals were the only locomotor group that had a sample size large enough for analysis.

### Dissection

Prior to dissection, specimen body mass was recorded. For specimens for which whole body masses were not measurable, body mass value were taken from Smith et al. [24]. Limbs were removed preferably with the skin still *in situ* on the limb. However, in some cases the specimen was skinned prior to dissection to accommodate needs of colleagues that donated the specimen to this study. Since we dissected limbs from the carcass with skin overlying the limb muscles, we used osteological markers to position the incisions necessary to remove the limb. Further description of limb dissection from the torso is provided in File S1.

### Measuring inertial properties

Prior to data collection all limbs were fully thawed, with the exception of limbs of *Equus caballus*. Detailed description of measurement of limb inertial properties are provided as supporting information in File S1; however, a brief overview follows. After initially weighing the limb, limb length was measured in its passively flexed position, after manually extending the limb and allowing it to passively flex on its own accord. Limb length was measured as the distance between the limb's pivot (e.g., the hip joint) and the distal extremity of the limb. The passively flexed length of the limb was chosen due to known postural differences between small and large mammals [25]–[26], with smaller and larger mammals having crouched and upright limb postures, respectively. Next, the limb was attached to a bar, with known inertial properties, and the limb's COM was found by hanging the limb-bar combination from two spring scales (Fig. S1 in File S1). After this, the limb and bar were fitted onto a pivot in line with the limb's axis of rotation, and the limb was then offset from the vertical by ∼20–30° and then released to swing freely about the pivot. During swinging, the limb was videotaped at 30 fps for larger mammals (e.g., antelope, wolves, foxes) and at 90–120 fps for smaller mammals (e.g., squirrels and chipmunk). Calculating the natural period of the limb-bar combination from the video, limb MOI, radius of gyration, and natural frequency could be calculated. A more detailed description of inertial property measurement is provided File S1, along with an assessment of measurement error (Table S1 in File S1). Values of inertial properties for each species are provided in Appendix A (Tables SA1 and SA2 in File S1).

### Determining scaling patterns

Scaling relationships are typically expressed as a power function of the form y = ax^b^, where ‘a’ is the allometric coefficient and ‘b’ is the allometric exponent [27]. One way to identify the biological import of scaling patterns is through comparisons of empirical data to the predictions of null model of geometric similarity [13]. Geometric similarity predicts that proportions remain constant with changes in body size (isometry). The predictions of geometric similarity also conform to how animals should theoretically scale in order to maintain constant stresses and deformations, resulting from maximal muscle forces, with changes in body size [28]. The allometric exponents predicted by geometric similarity for limb length and all inertial properties are listed in Table 2 and derivations for predicted exponents are provided in File S1.

**Table 2:** Exponents predicted by geometric similarity for limb length and each of the inertial properties measured in this study.

Inertial Property	Definition	Prediction
Length	Distance from the limb's pivot to its distal most ungual	1/3
Mass	Mass of the entire limb musculoskeletal system	1.0
Center of Mass (COM) position	Centroid of limb mass distribution along its proximo-distal length	1/3
Moment of Inertia (MOI)	Resistance of the limb to swinging about its pivot. MOI is determined by mass and shape	1.67
Radius of Gyration	The shape component of MOI along the limb's proximo-distal length	1/3
Natural Frequency	Optimal frequency of oscillation for a limb as it swings about its pivot	−1/6

Predicted exponents represent the exponent b in the relationship y = a(X)^b^, where x equals body mass and y represents a given inertial property.

## Statistics

### Regressions

Using species means, scaling patterns were assessed using Reduced Major Axis (RMA or Model II) bivariate regression. Prior to analysis, all data was log_10_-transformed. The following function describes the regression line: log(y)  =  b(log(x)) + log(a), the log-transformed version of the general allometric equation y = ax^b^. RMA bivariate regressions were chosen to examine the scaling of inertial properties since Model II regression assumes that neither variable is independent in the strict sense and that both variables contain some degree of error (either measurement errors and/or biological variation) [29]–[31]. Model II regressions are also appropriate for comparing regression slopes to theoretical values [32].

We used two tests for departures from null model predictions, one using effect size statistics and the other using null hypothesis significance testing. The regression slope was our measure of effect size, and we generated 95% confidence intervals for the slope to determine its relation to null model predictions [33]. If the 95% confidence interval excluded the null model predicted value, then the departure from geometric similarity was considered significant. For null hypothesis significance testing, we performed F-tests to compare each regression to null model predictions. Differences between the slope and predicted value were significant if *P*<0.05. RMA regressions and F-tests were performed in R [34] using the modules lmodel2 and SMATR [32], [35]. Instead of performing a Bonferroni correction for our several regressions, we instead present the results of both our measure of effect size and our null hypothesis significance testing. Effect size measures convey biological significance and meaning, whereas null hypothesis testing conveys only statistical significance [33], [36]. Moreover, while Bonferroni corrections guard against increased Type I errors, they are prone to increased Type II errors (e.g., decreased statistical power). As an alternative to Bonferroni corrections, we follow the recommendations of Nakagawa [36] and present the result of both effect size measures and the result of null hypothesis significance testing. In no instance do the results of our two tests for null model departures disagree with one another. Consequently for succinctness, we only report the *P*-values of F-tests in the text of the Results and Discussion sections.

### Comparing fore- and hindlimb scaling

To test for significant differences in the scaling of fore- and hindlimbs, we performed tests for common slope and elevation. The common slope test is a likelihood ratio test, and the test for common elevation is a Wald test. Both likelihood ratio and Wald tests were performed using SMATR [32].

### Comparative methods

Many of the taxa present in this study are closely related, being nested within sub-clades of Mammalia (e.g., canids). The limb morphologies of these taxa were inherited from proximate, common ancestors and are not independently derived in each species, making conventional statistical methods inappropriate for inferring trait evolution [37]. To take this non-independence into account when inferring the diversification of traits, we first tested for phylogenetic signal – the tendency for phenotypic similarity to increase with phylogenetic relatedness [38] – by calculating Pagel's lambda (λ: [39]–[40]), an effect size measure of phylogenetic signal [41]. For λ, a value of 0.0 indicates that all traits evolved independently among taxa (i.e., no phylogenetic influence on the data). A value of 1.0 indicates that the traits have evolved under Brownian motion along the specified branches of the phylogeny (i.e., shared ancestry reflects phenotypic diversity). We then performed generalized least squares (GLS) regressions of limb length and each inertial property against body mass and tested for phylogenetic signal amongst the residuals [42]. Testing for phylogenetic signal within residuals reveals whether phylogeny has influenced the covariance amongst traits, as the presence of phylogenetic signal in one or two individual traits does not necessarily entail phylogenetic signal in their covariation [42]. Tests for phylogenetic signal were performed with modules ape [43] and pmc [44] in R. Upon finding significant phylogenetic signal within residuals, regressions were performed an additional time using phylogenetic generalized least squares (PGLS) regressions that simultaneously estimate Pagel's λ alongside other regression statistics (R code provided in [42]).

The taxa included in this study represent a diverse sample, spanning eight taxonomic orders. In order to calculate independent contrasts, a composite phylogeny had to be constructed (Fig. 3). Relationships between orders and families were primarily based upon [45], whereas topologies of mammalian families were based upon phylogenies focusing on a single order or family. A list of published phylogenies used to construct the composite phylogeny for this study is available in File S1. Branch lengths were treated two different ways: 1) setting branch lengths to divergence times and 2) setting all branch lengths equal to 1.0 (unity). Phylogenies used to scale divergence times in our composite phylogeny are listed in Table S2 in File S1. Setting all branches to equal length forces traits to change at branching points and represents a punctuated model of evolution [46]. All trees were constructed in Mesquite [47].

Note that we had two goals in our study of limb inertial properties: to understand comparative function and inferred locomotor performance and to ascertain trait diversification across quadrupedal mammals. Though the testing of phylogenetic signal and performing λ-regressions is essential for understanding trait diversification, the raw RMA regressions are still crucial for understanding differences in comparative function and locomotor performance amongst the present day species (i.e., those species forming the tips of the phylogeny). So while these two sets of analyses certainly shed light on one another, it should be borne in mind that each one served a distinct goal.

## Results

### Limb length

**Figure 4:**
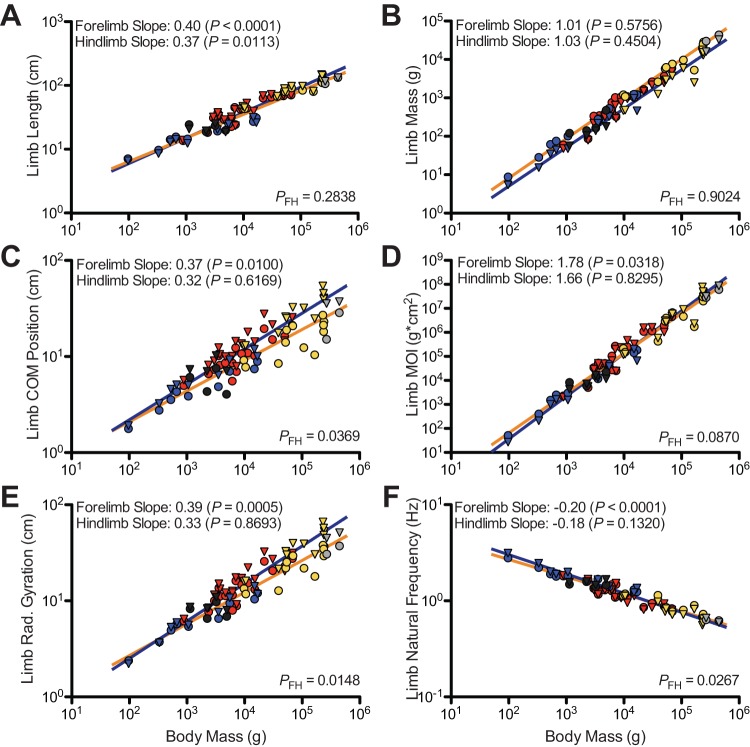
Bivariate plots of limb traits scaled against body mass for the entire mammalian sample. The scaling of limb length is depicted in A, limb mass in B, and COM position in C. The scaling of limb MOI is portrayed in D, radius of gyration in E, and natural frequency in F. Triangles and blue trend lines denote forelimbs, whereas circles and orange trend lines denote hindlimbs. Carnivorans are represented by red points, rodents by blue, artiodactyls by yellow, and perissodactyls by grey. *P*-values for slopes indicate departures from geometric similarity when *P*<0.05. When *P*_FH_<0.05, differences in fore- and hindlimb slope are significant. Note not all axes to the same scale.

**Figure 5:**
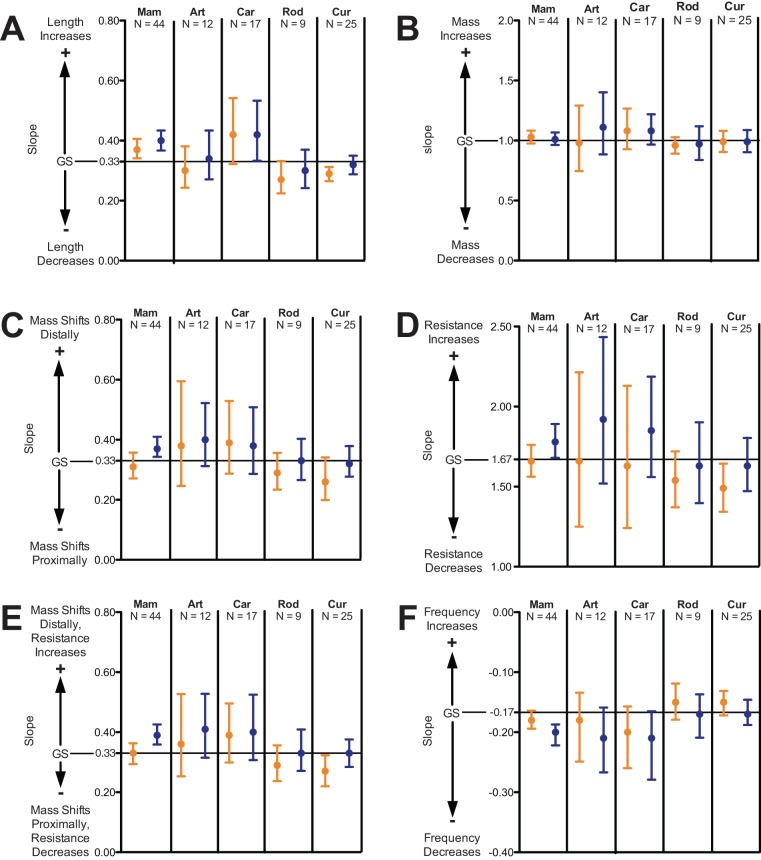
Slopes of regressions for Mammalia and the mammalian subgroups separately analyzed in this study. Slopes for limb length are shown in A, whereas slopes for limb inertial properties are shown in B to F. ‘Mam’ denotes our whole sample for Mammalia, and, among taxonomic subgroups, ‘Car’ denotes Carnivora, ‘Art’ denotes Artiodactyla, and ‘Rod’ denotes Rodentia. For locomotor subgroups, ‘Cur’ denotes cursors. Each slope estimate is plotted with its 95% confidence limits (whiskers). Blue denotes forelimbs, whereas orange denotes hindlimbs. The horizontal line in each plot represents the slope predicted by geometric similarity (GS). The slope cannot be distinguished from the null model prediction when confidence limits include the predicted value. Positive and negative allometry occur when the predicted values respectively lie below or above slope confidence limits. Positive allometry is denoted ‘+’, while negative allometry is denoted by ‘-’. Note not all axes to the same scale.

Both fore- and hindlimb length are positively allometric with respect to body mass, (*P*<0.05) with slopes of 0.40 and 0.37, respectively (Figs. 4A and 5A; Table 3). For mammalian subgroups, forelimb slopes range from 0.30 (Rodentia) to 0.42 (Carnivora), while hindlimb slopes range from 0.27 (Rodentia) to 0.42 (Carnivora). Apart from cursorial mammals, fore- and hindlimb slopes for each of the subgroups have wide confidence limits, likely due in part to their smaller sample sizes. For each of these groups, the slopes do not significantly depart from geometric similarity (*P*>0.05). For cursors, forelimb length is isometric with body mass, while hindlimb length is negatively allometric with body mass.

**Table 3:** Results for regressions of limb length against body mass.

Taxon/Group	N	Intercept	Slope	95% C. L.	R^2^	*P*	Departures?
**Hindlimbs**							
Mammalia	44	0.07	0.37	0.342, 0.408	0.9203	<0.0001	**0.0113**
Artiodactyla	12	0.43	0.30	0.243, 0.381	0.8966	<0.0001	0.3386
Carnivora	17	−0.08	0.42	0.322, 0.542	0.7705	<0.0001	0.0845
Rodentia	9	0.34	0.27	0.224, 0.332	0.9507	<0.0001	0.0481
Cursors	25	0.51	0.29	0.265, 0.313	0.9629	<0.0001	**0.0015**
**Forelimbs**							
Mammalia	44	−0.02	0.40	0.367, 0.434	0.9262	<0.0001	**<0.0001**
Artiodactyla	12	0.28	0.34	0.271, 0.434	0.8858	<0.0001	0.7891
Carnivora	17	−0.07	0.42	0.333, 0.533	0.8136	<0.0001	0.0509
Rodentia	9	0.24	0.30	0.242, 0.370	0.9433	<0.0001	0.2750
Cursors	25	0.40	0.32	0.288, 0.350	0.9488	<0.0001	0.3187

Values in the ′95% C. L.’ provide the 95% confidence interval for each slope. *P*-values in the column ‘Departures?’ are the results of F-tests testing for departures from the predicted slope of 1/3. Significant results for F-tests are *P*<0.05 and are highlighted in bold.

Differences in slope between regressions for fore- and hindlimb length upon body mass are not significant for each group studied (*P*>0.05). Likewise, differences in trendline elevation are also not significant between fore- and hindlimbs for each group studied (*P*>0.05).

### Limb mass

The slopes for fore- and hindlimb mass for Mammalia are 1.01 and 1.03, respectively (Figs. 4B and 5B; Table 4), with limb mass scaling isometrically with body mass (*P*>0.05). Likewise, for all the subgroups sampled, the scaling of fore- and hindlimb mass parallels the scaling relationships determined for Mammalia. Among the subgroups, the scaling of limb mass does not deviate from the predictions of geometric similarity for either fore- or hindlimbs (*P*>0.05 for all groups).

**Table 4:** Results for regressions of limb mass against body mass.

Taxon/Group	N	Intercept	Slope	95% C. L.	R^2^	*P*	Departure?
**Hindlimbs**							
Mammalia	44	−1.15	1.03	0.970, 1.069	0.9761	<0.0001	0.4504
Artiodactyla	12	−0.92	0.98	0.745, 1.291	0.8434	<0.0001	0.7683
Carnivora	17	−1.42	1.08	0.928, 1.266	0.9196	<0.0001	0.2886
Rodentia	9	−0.90	0.96	0.891, 1.028	0.9936	<0.001	0.1908
Cursors	25	−0.92	0.98	0.907, 1.053	0.9701	<0.0001	0.5389
**Forelimbs**							
Mammalia	44	−1.32	1.01	0.964, 1.067	0.9734	<0.0001	0.5756
Artiodactyla	12	−1.87	1.11	0.885, 1.401	0.8922	<0.0001	0.3236
Carnivora	17	−1.52	1.08	0.966, 1.219	0.9552	<0.0001	0.1570
Rodentia	9	−1.17	0.97	0.838, 1.119	0.9737	<0.0001	0.6148
Cursors	25	−1.19	0.99	0.902, 1.087	0.9530	<0.0001	0.8248

Values in the ′95% C. L.’ provide the 95% confidence interval for each slope. *P*-values in the column ‘Departures?’ are the results of F-tests testing for departures from the predicted slope of 1.0. All three null models predict a slope of 1.0. Significant results for F-tests are *P*<0.05 and are highlighted in bold.

For each of the groups sampled, there is no significant difference in slopes (*P*>0.05) between hindlimbs and forelimbs. With regards to trendline elevation, a Wald's test confirms that intercepts are significantly different between hindlimbs and forelimbs (*P*<0.05) for each group sampled. The results of the Wald's tests indicate that for mammals of a given body mass, hindlimbs have a greater mass than forelimbs.

### Limb center of mass (COM) position

Forelimb COM position was measured relative to the dorsal extreme of the scapular spine, whereas hindlimb COM position was the distance was measured relative to the hip joint (for detailed description of dissection protocol, see supporting information in File S1). With a slope of 0.37, forelimb COM position scales via positive allometry (*P* = 0.0100; Figs. 4C and 5C; Table 5), indicating that forelimb COM position shifts relatively distally with increasing body mass. With a slope of 0.31, hindlimb COM position scales isometrically with body mass (*P* = 0.3207). Slopes for mammal subgroups range from 0.26 (cursors) to 0.37 (Carnivora). For all subgroups, fore- and hindlimb COM position scales according to geometric similarity (*P*>0.05), indicating that limb mass distribution remains unchanged with respect to increasing body mass. For Carnivora, the slope is consistent with geometric similarity (*P* = 0.4088). However, the confidence limits for some of the slopes are quite large (e.g., Artiodactyla), again due to limited sample size.

**Table 5:** Results for regressions of limb COM position against body mass.

Taxon/Group	N	Intercept	Slope	95% C. L.	R^2^	*P*	Departures?
**Hindlimbs**							
Mammalia	44	−0.32	0.32	0.276, 0.361	0.8150	<0.0001	0.6269
Artiodactyla	12	−0.64	0.37	0.234, 0.587	0.5440	0.0062	0.5132
Carnivora	17	−0.45	0.37	0.284, 0.485	0.7578	<0.0001	0.4088
Rodentia	9	−0.28	0.29	0.234, 0.356	0.9444	<0.0001	0.1555
Cursors	25	−0.04	0.26	0.200, 0.336	0.6333	<0.0001	0.0572
**Forelimbs**							
Mammalia	44	−0.40	0.37	0.343, 0.410	0.9183	<0.0001	**0.0100**
Artiodactyla	12	−0.53	0.40	0.312, 0.522	0.8626	<0.0001	0.1306
Carnivora	17	−0.39	0.38	0.286, 0.508	0.7196	<0.0001	0.3380
Rodentia	9	−0.30	0.33	0.266, 0.403	0.9452	<0.0001	0.8499
Cursors	25	−0.14	0.32	0.277, 0.379	0.8668	<0.0001	0.7285

Values in the ′95% C. L.’ provide the 95% confidence interval for each slope. *P*-values in the column ‘Departures?’ are the results of F-tests testing for departures from the predicted slope of 1/3. Significant results for F-tests are *P*<0.05 and are highlighted in bold.

For Mammalia, fore- and hindlimbs significantly differ in slope (*P* = 0.0369), with forelimbs having a greater slope than hindlimbs. Within each mammalian subgroup, fore- and hindlimbs do not significantly differ in slope (*P*>0.05) but significantly differ in trendline elevation (*P*<0.05). Forelimbs have a greater intercept than hindlimbs in all subgroups, except for Artiodactyla and Carnivora, although for each subgroup the magnitude of these differences is small (≤0.10).

### Limb moment of inertia (MOI)

The slope for forelimb MOI is 1.78, which significantly differs from the prediction of geometric similarity (P<0.05; Figs. 4D and 5D; Table 6). The slope for hindlimb MOI is 1.66. Testing the slope against geometric similarity's prediction finds that the slope cannot be distinguished from this value (*P* = 0.8295). Among mammalian subgroups, forelimb slopes ranged from 1.63 (cursors) to 1.92 (Artiodactyla), and hindlimb slopes ranged from 1.49 (cursors) to 1.82 (Carnivora). Fore- and hindlimb slopes, which by and large exhibit large confidence limits, do not significantly differ from the prediction of geometric similarity. The sole exception of this, is the slope for cursorial mammal hindlimb MOI, which is significantly less than 1.67 and, consequently, negatively allometric.

**Table 6:** Results for regressions of limb MOI against body mass.

Taxon/Group	N	Intercept	Slope	95% C. L.	R^2^	*P*	Departures?
**Hindlimbs**							
Mammalia	44	−1.51	1.66	1.562, 1.762	0.9627	<0.0001	0.8295
Artiodactyla	12	−1.57	1.66	1.250, 2.215	0.8304	<0.0001	0.9780
Carnivora	17	−2.05	1.82	1.525, 2.168	0.8966	<0.0001	0.3215
Rodentia	9	−1.18	1.54	1.371, 1.720	0.9839	<0.0001	0.1235
Cursors	25	−0.63	1.49	1.343, 1.644	0.9449	<0.0001	**0.0254**
**Forelimbs**							
Mammalia	44	−2.02	1.78	1.680, 1.891	0.9640	<0.0001	**0.0318**
Artiodactyla	12	−2.76	1.92	1.519, 2.434	0.8861	<0.0001	0.2143
Carnivora	17	−2.13	1.85	1.560, 2.187	0.9049	<0.0001	0.2242
Rodentia	9	−1.66	1.63	1.397, 1.902	0.9701	<0.0001	0.7223
Cursors	25	−1.25	1.63	1.472, 1.804	0.9444	<0.0001	0.6213

Values in the ′95% C. L.’ provide the 95% confidence interval for each slope. *P*-values in the column ‘Departures?’ are the results of F-tests testing for departures from the predicted slope of 5/3 (1.67). Significant results for F-tests are *P*<0.05 and are highlighted in bold.

Slopes for forelimbs do not significantly differ from slopes for hindlimbs for Mammalia (*P* = 0.0870) or any of the subgroups sampled (*P*>0.05), in spite of differing results between fore- and hindlimbs (i.e., isometry vs. allometry) when comparing fore- and hindlimb slopes to null model predictions separately. When testing for differences in trendline elevation between fore- and hindlimbs, we found no significant differences between fore- and hindlimbs for any sampled group (*P*>0.05).

### Limb radius of gyration

For the entire mammalian sample, the slopes for the radius of gyration are 0.39 and 0.33 for fore- and hindlimbs, respectively (Figs. 4E and 5E; Table 7). The slope for the hindlimb does not significantly deviate from geometric similarity (*P* = 0.7222), which indicates that the hindlimb's radius of gyration remains constant relative to body mass with increasing body size. However, the slope for the forelimb does significantly deviate from the null model (*P* = 0.0005), being positively allometric. Thus for the entire mammalian sample, as body mass increases the forelimb's radius of gyration shifts relatively distally along the limb. Among the mammalian subgroups studied, forelimb slopes ranged from 0.33 (Rodentia and cursors) to 0.41 (Artiodactyla), whereas hindlimb slopes ranged from 0.27 (cursors) to 0.39 (Carnivora). Across all subgroups, forelimb slopes do not significantly differ from 0.33 (*P*>0.05), the slope predicted by geometric similarity. Likewise, hindlimb slopes also do not significantly depart from geometric similarity apart from one exception. In cursorial mammals, hindlimb radius of gyration is negatively allometric with body mass (*P* = 0.0249), having a slope of 0.27 and indicating that the hindlimb's radius of gyration shifts proximally along the limb with increasing body size.

**Table 7:** Results for regressions of limb radius of gyration against body mass.

Taxon/Group	N	Intercept	Slope	95% C. L.	R^2^	*P*	Departures?
**Hindlimbs**							
Mammalia	44	−0.23	0.33	0.297, 0.366	0.8884	<0.0001	0.8693
Artiodactyla	12	−0.41	0.36	0.246, 0.529	0.6908	0.0008	0.6583
Carnivora	17	−0.39	0.39	0.299, 0.496	0.7826	<0.0001	0.2441
Rodentia	9	−0.15	0.29	0.237, 0.356	0.9479	<0.0001	0.1586
Cursors	25	0.08	0.27	0.222, 0.323	0.8058	<0.0001	**0.0249**
**Forelimbs**							
Mammalia	44	−0.38	0.39	0.359, 0.426	0.9241	<0.0001	**0.0005**
Artiodactyla	12	−0.46	0.41	0.315, 0.528	0.8640	<0.0001	0.1103
Carnivora	17	−0.38	0.40	0.307, 0.525	0.7562	<0.0001	0.1610
Rodentia	9	−0.26	0.33	0.271, 0.409	0.9462	<0.0001	0.9952
Cursors	25	−0.06	0.33	0.284, 0.376	0.8947	<0.0001	0.7898

Values in the ′95% C. L.’ provide the 95% confidence interval for each slope. *P*-values in the column ‘Departures?’ are the results of F-tests testing for departures from the predicted slope of 1/3. Significant results for F-tests are *P*<0.05 and are highlighted in bold.

For Mammalia, the slope for forelimbs is significantly greater than hindlimb slope (*P* = 0.0148). For the subgroups, within-group differences in fore- and hindlimb slope are not significant (*P*>0.05). Among the subgroups, forelimb intercepts are significantly greater than hindlimbs intercepts in Artiodactyla, and cursors (*P*<0.05), indicating that in these clades the center of gyration is placed more distally in the forelimb than in the hindlimb. In Rodentia and Carnivora, intercepts did not significantly differ between fore- and hindlimb (*P*>0.05).

### Limb natural frequency

For Mammalia, slopes for the scaling of natural frequency are −0.20 and −0.18 for fore- and hindlimbs, respectively (Figs. 4F and 5F; Table 8). The forelimb's slope significantly departs from geometric similarity (*P*<0.0001), while the hindlimb's does not (*P* = 0.1320). Thus forelimb natural frequency scales with negative allometry, whereas hindlimb natural frequency scales isometrically. Among the sampled subgroups, forelimb slopes ranged from a high value of −0.21 (Artiodactyla and Carnivora) to a low value of −0.17 (Rodentia and cursors). Hindlimb slopes ranged from −0.20 (Carnivora) to −0.15 (Rodentia and cursors). For each group, the fore- and hindlimb slopes are consistent with the predictions of geometric similarity (*P*>0.05).

**Table 8:** Results for regressions of limb natural frequency against body mass.

Taxon/Group	N	Intercept	Slope	95% C. L.	R^2^	*P*	Departures?
**Hindlimbs**							
Mammalia	44	0.79	−0.18	−0.194, −0.164	0.9247	<0.0001	0.1320
Artiodactyla	12	0.82	−0.18	−0.249, −0.134	0.7983	<0.0001	0.5403
Carnivora	17	0.87	−0.20	−0.260, −0.157	0.7852	<0.0001	0.1295
Rodentia	9	0.70	−0.15	−0.179, −0.119	0.9479	<0.0001	0.1616
Cursors	25	0.64	−0.15	−0.172, −0.131	0.8993	<0.0001	0.1187
**Forelimbs**							
Mammalia	44	0.88	−0.20	−0.222, −0.187	0.9234	<0.0001	**<0.0001**
Artiodactyla	12	0.89	−0.21	−0.267, −0.159	0.8627	<0.0001	0.0992
Carnivora	17	0.90	−0.21	−0.279, −0.165	0.7669	<0.0001	0.0607
Rodentia	9	0.80	−0.17	−0.209, −0.137	0.9429	<0.0001	0.8815
Cursors	25	0.69	−0.17	−0.188, −0.146	0.9138	<0.0001	0.9074

Values in the ′95% C. L.’ provide the 95% confidence interval for each slope. *P*-values in the column ‘Departures?’ are the results of F-tests testing for departures from the predicted slope of −1/6 (−0.167). Significant results for F-tests are *P*<0.05 and are highlighted in bold.

For Mammalia as a whole, the slope for fore- and hindlimbs significantly differ (*P* = 0.0267). However, the slopes for fore- and hindlimbs do not differ for each mammalian subgroup (*P*>0.05). With regards to trendline elevation, forelimb y-intercepts are significantly greater than those of the hindlimb (*P*<0.05) in all subgroups except Rodentia. However, the magnitude of these differences is small (<0.1). In Rodentia, there is no significant difference in trendline elevation between fore- and hindlimbs.

### Phylogenetic signal within limb inertial properties

Each individual trait studied exhibited significant phylogenetic signal by having confidence limits that exclude a value of 0.0 (Tables S3–S4 in File S1), regardless of how branch lengths are scaled. However, whether a trait evolves according to a Brownian motion model of evolution, with its confidence interval for λ including a value of 1.0, depends on the method of scaling branch lengths. When scaling branch lengths to divergence times, confidence limits did not include 1.0, with the exception of forelimb natural frequency. Yet when branch lengths are scaled to unity, the upper confidence limit for each trait is 1.0 (which is the upper bound for λ in the pmc package [43]). When scaling branch lengths to divergence times and unity, residuals from regressions of limb length against body mass possessed significant phylogenetic signal (Table S3 in File S1). The confidence limits for divergence time-scaled branch lengths exclude 1.0, while unity-scaled branch lengths include this value. When scaling branch lengths to unity, residuals for COM position, and radius of gyration contain significant phylogenetic signal (Table S4 in File S1). However, confidence limits for these two traits exclude a value 1.0 (though upper limits approach 1.0).

The result of λ-regressions are in Table S5 in File S1. When scaling branch lengths to divergence times, PGLS λ-regressions for forelimb length against body mass yield a slope of 0.33 (0.297, 0370) and a λ-value of 0.90 (0.799, 1.010). PGLS λ-regressions for hindlimb length against body mass yield a slope of 0.30 (0.266, 0.337) and a λ-value of 0.93 (0.839, 1.018). When scaling branch lengths to unity, the regression slope for forelimb length is 0.32 (0.285, 0.351), while that for the hindlimb is 0.29 (0.258, 0.325). For these regressions, λ is 1.00 for both fore- and hindlimbs. For λ-regressions of COM position and radius of gyration, slope values are respectively 0.33 and 0.34 for forelimbs and respectively 0.27 and 0.29 for hindlimbs. Among COM position and radius of gyration scaling, the only instance of allometry is hindlimb COM position scaling, for which the confidence limits are (0.220, 0.323). λ-values for these regressions range from 0.62 to 0.73, with all confidence limits indicating a Brownian motion model of trait co-evolution (i.e., confidence limits inclusive of 1.0).

## Discussion

### Scaling limb inertial properties to body size

For Mammalia, the scaling of hindlimb inertial properties largely fit the geometric similarity model, while the scaling of several forelimb inertial properties depart from the null model (Figs. 4 and 5; Tables 4, 5, 6, 7, and 8). For both fore- and hindlimbs, limb mass scales isometrically with body mass, and limb length scales with positive allometry. Thus, smaller and larger-bodied mammals possess limbs having the same proportion of body mass; however, larger mammals possess disproportionately longer limbs. The scaling of hindlimb COM position, radius of gyration, and natural frequency are consistent with the geometric similarity model. However, the scaling of forelimb COM position and radius of gyration did not conform to geometric similarity, instead being positively allometric. Relative to the predictions of geometric similarity, forelimbs became distally heavier relative to body mass as body size increased in mammals.

MOI is a function of both mass and radius of gyration, and Equation 1 can be rewritten as:
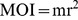
(2)


with *m* being limb mass and *r* being limb radius of gyration. In the hindlimb, MOI scales according to geometric similarity; this is due to both hindlimb mass and radius of gyration scaling isometrically with body mass. The positive allometry of forelimb MOI is due to a combination of isometry of forelimb mass (M^1.01^) and positive allometry of forelimb radius of gyration (M^0.39^):
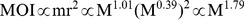
(3)


The estimated exponent of 1.79 in Equation 3 closely approximates the value of 1.78 found when regressing forelimb MOI (including the scapula) against body mass.

The negative allometry of forelimb natural frequency is the result of positive allometry of the forelimb COM position, MOI, and radius of gyration. Increases in MOI result in a decrease in natural frequency (Eq. S5 in File S1). By substituting Equation 2 into Equation S5, the following expression can be obtained:
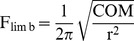
(4)


Equation 4 shows that increases in the radius of gyration result in decreases in natural frequency. COM position and radius of gyration both scale with positive allometry, however, given that the radius of gyration is a squared term in Equation 4, it has a strong effect upon the scaling of natural frequency.

The positive allometry of forelimb COM position, MOI, and radius of gyration, as well as the isometry of hindlimb inertial properties, is likely dictated by the functions of fore- and hindlimbs. During steady-state locomotion, hindlimbs function to provide a net propulsive force (in terms of fore-aft forces) and positive work [48]–[51], whereas forelimbs provide a net braking force and negative work [48]–[51]. Concentrating hindlimb mass about the hip likely improves the hindlimb's ability to generate thrust and positive work.

The forelimb's role in decelerating the body during locomotion may not require a proximal concentration of mass. Recent studies have started to investigate whether the forelimb functions as a strut during steady-state locomotion, with the ground reaction force being oriented through the forelimb's pivot [52]–[54]. As the ground reaction force passes through or near the forelimb's pivot, the thoracic extrinsic muscles do not actively retract the limb during stance. In light of this, the muscle mass of the forelimb may not need to be concentrated proximally. However, while electromyographic data is consistent with forelimbs acting as struts during steady-state locomotion [52]–[54], further data on foot center of pressure, shoulder joint identification, and segment inclusive inverse dynamics are needed to definitively address whether the forelimb acts as a strut (Hutchinson, pers. comm.). Also, as the forelimb typically supports 60% of total body weight [55], there might be a need for relatively larger muscles acting about the distal joints to counter the relatively larger vertical component of the ground reaction force acting on the forelimb. While positive allometry of forelimb COM position, MOI, and radius of gyration should increase the cost of swinging the limb between small and large mammals, it theoretically may have a negligible effect on the potential for some aspects of locomotor performance (see below in ‘*The potential for angular acceleration*’).

However, during accelerative locomotion, forelimbs act as levers and generate a net propulsive force, as do hindlimbs during both accelerative and steady-state locomotion [56]–[57]. The lever-like function of the forelimb is accomplished via large extrinsic muscles, such as the *latissimus dorsi*. The forelimb extrinsic muscles are concentrated away from the forelimb itself, originating from the body wall (we cut these muscles away from the limb during dissection of the forelimb). Thus, our analysis of the scaling of forelimb inertial properties is likely a better reflection of a ‘strut-like’ forelimb, which may be more in line with how the forelimb functions during steady-state locomotion.

### Geometric Similarity

Apart from maintaining constant proportions, the geometric similarity model has recently been proposed to describe how bodily proportions respond to muscles exerting their maximum force. Scaling relationships derived by Norberg & Wetterholm Aldrin [28] to maintain constant axial and shear stress, as well as scaling relationships to maintain constant deformations due to bending and torsion, all converged upon the geometric similarity model. The authors therefore viewed geometric similarity not only as a model for maintaining constant proportions with changes in size, but also as a model for equal resistance to muscles exerting maximum forces with changes in size. If the argument proposed by Norberg & Wetterholm Aldrin is correct, then the hindlimb musculoskeletal system may have evolved in response to muscles exerting their maximum forces. Given the hindlimb's role in producing thrust and positive work in both accelerative and non-accelerative locomotion [56]–[58], it would not be surprising that resistance to muscles exerting maximum force guides the relationship between body size and whole hindlimb morphology. The finding that forelimb inertial properties do not scale according to geometric similarity suggests that the loads imposed by forelimb muscles exerting maximum force is likely not the factor dominating how forelimb inertial properties scale with body mass across terrestrial mammals.

### Phylogenetic signal and regression residuals

There is significant phylogenetic signal within each of the individual traits studied (Tables S2 to S4 in File S1). While this result holds regardless of whether branch lengths are scaled to divergence times or unity, the results slightly differ according to which method of scaling branch lengths. Scaling branch lengths to unity yields all traits evolving according a model of Brownian motion (confidence limits include a value of 1.0). In contrast, scaling branch lengths to divergence times uncovers only forelimb natural frequency as following a Brownian motion of evolution.

Therefore, shared evolutionary history among species influences the morphological design of their limbs (at least in terms of length and inertial properties). However, it is possible that this result is dependent upon the taxonomic sampling of our study. Several of our sampled subclades possess a monotypic limb morphology, such as Artiodactyla and Canidae, and many members of these subclades occupy similar ecological niches. Though we sampled more functionally and ecologically diverse subclades, such as Rodentia, we were not able to sample them as extensively as the more monotypic subclades. Additionally, many of our sampled sampled species across subclades are cursors – which possess numerous morphological traits advantageous for locomotion whether through shared ancestry or convergence [59]–[61]. However, given that many subclades in fact possess monotypic limb morphology, our results may still remain in light of more extensive taxonomic sampling. To better determine to what extent mammlian limb morphological traits are influenced by morphology, a greater depth of sampling both in terms of taxonomy and function is necessary.

Applying to both methods of branch length scaling, we found significant phylogenetic signal within co-variation of limb length alongside body mass, indicating that shifts in body mass along a lineage were accompanied by shifts in limb length. The results of λ-regressions reveal that body mass and limb length tend to increase and decrease together. This result suggests that for mammals to function in a terrestrial environment, limbs can be neither too long nor too short relative to body mass. In line with this notion is that long limbed mammals tend to favor pacing gaits, allegedly in part to minimize the chance of ipsilateral limbs interfering with one another during locomotion [62]–[64], though few mammals outside of Old World camelids actually use pacing gaits [65]–[66]. On the other hand, limbs that are too short relative to body mass may limit the ability of mammals to navigate uneven terrain or may only allow strides of insufficient length relative to body mass. When only scaling branch lengths to unity, we found significant phylogenetic signal in COM position and radius of gyration, suggesting that these traits may better follow a model of rapid trait evolution. This possibility should be explored more in depth in future studies exploring differing methods of scaling branch lengths and models of branch length transformation [39].

**Figure 6:**
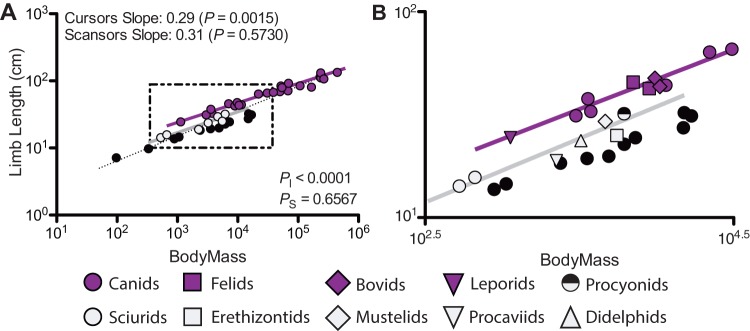
The scaling of hindlimb length to body mass in cursorial and scansorial taxa. The position of cursors (purple) and scansors (grey) relative to the overall mammalian trend is shown in A, with the dashed line being the trend line for the entire mammalian sample. B corresponds to area of the plot where cursors and scansors overlap in body mass, bordered by the dashed lines in A. *P*-values for slopes indicate departures from geometric similarity when *P*<0.05. When *P*_I_<0.05 and *P*_S_<0.05 indicate significant differences between intercepts and slopes, respectively, between cursors and scansors.

The λ-regressions of limb length vs. body mass disagree with the RMA regressions, finding instead that limb length scales according to geometric similarity (Table S5 in File S1). The discrepancy in results between non-phylogenetic and phylogenetic regressions may stem from differences in scale effects between different subclades (i.e., branches of the tree) and locomotor subgroups of mammals (Table 9), some of which exhibit distinct morphologies. Comparing the slopes and intercepts for Artiodactyla, Carnivora, and Rodentia – the three mammalian orders with the largest samples – forelimbs share a common slope yet significantly differ in intercept, while hindlimbs significantly differ in slope. Thus among individual monophyletic groups within Mammalia, forelimbs appear to differ in absolute length (i.e., intercepts) while exhibiting similar scale effects (i.e., slopes). In contrast, among monophyletic groups, hindlimbs exhibit differing scale effects. However, when comparing cursorial and scansorial mammals, the two locomotor groups with the largest sample sizes in our study, a more interesting result emerges (Fig. 6A). Both fore- and hindlimbs exhibit similar scale effects (i.e., statistically indistinguishable slopes), indicating that between these two locomotor groups body size has a similar influence upon limb length. However, cursors and scansors differ in the absolute length of their limbs (i.e. intercepts), with cursors having longer limbs for a given body mass than scansors (Fig. 6B). While this finding is restricted to only two locomotor groups, the results suggest that differences in functional and ecological specializations entail changes in the overall design of whole limbs irrespective of body size. More specifically, the results suggest that ecological or functional specializations are not occurring due to a differential influence of body size among individual specializations (though cursors attain overall larger body sizes; Fig. 6A). In light of these results, it also seems possible that the overall scaling trends for terrestrial mammals as a whole may be driven by the differences in overall whole limb design between taxa of differing limb specializations. However, the difference in sample size between cursors (N = 25) and scansors (N = 7) should be noted, as this may have a strong bearing on these results. To adequately test how robust our finding is and to definitively examine whether this finding applies to all locomotor groups, more extensive sampling of the differing mammalian locomotor groups (i.e, fossorial, natatorial, scansorial taxa) is required.

**Table 9:** Comparisons of limb length scaling trends between taxonomic (Artiodactyla, Carnivora, and Rodentia) and locomotor (cursors and scansors) subgroups of Mammalia.

	Group	Slope	Diff_Slopes_	Intercept	Diff_Intercept_
Forelimbs	Artiodactyla	0.34	0.0830	0.28	<0.0001
	Carnivora	0.42		−0.07	
	Rodentia	0.30		0.24	
Hindlimbs	Artiodactyla	0.30	0.0301	0.45	–
	Carnivora	0.42		−0.08	
	Rodentia	0.27		0.35	
Forelimbs	Cursors	0.32	0.4285	0.40	<0.0001
	Scansors	0.35		0.16	
Hindlimbs	Cursors	0.29	0.6567	0.51	<0.0001
	Scansors	0.31		0.30	

Under ‘Diff_Slopes_’ the results of common slope tests are given, whereas under ‘Diff_Intercepts_’ the results of common elevation tests are given. Both sets of tests, the differences in slope and intercept were significant if *P*<0.05, and both sets of tests were performed using the R module SMATR [32].

### The scaling of limb length and locomotor costs

Due to the positive allometry of limb length, disproportionately longer limbs of larger mammals allow them to take longer strides and use a lower number of strides to cover a given distance than small mammals [16], [19],[67]. Given that the mass-specific metabolic energy consumed per stride is constant across small and large mammals [14], [17]–[18], the higher number of strides per unit distance of small mammals results in them having a higher mass-specific COT compared to large mammals. Thus, the limb lengths of large mammals may partly underlie the COT scaling [68], as larger mammals have longer limbs not only in terms of absolute length, but also relative to their body size. Furthermore, the scaling of limb length may also explain the scaling of stride length, which is positively allometric and scales as M^0.38^ at the trot-gallop transition speed [67]. This exponent closely matches the allometric exponents for the scaling of fore- (M^0.40^) and hindlimb length (M^0.37^). While the scaling of limb length may partly underlie how the cost of transport scales relative to body size, the scaling of other limb inertial properties (e.g., limb mass, MOI, etc.) do not appear to scale in such a manner as to reduce locomotor costs (e.g., forelimb MOI). However, any advantages that the positive allometry of limb length might confer to lower locomotor costs would also act in unison with advantages offered by scaling of other locomotor traits, such as the positive allometry of effective mechanical advantage [26] and the potential for elastic energy storage [69], or scale effects in mitigating collisional energy loss [20].

### Limb inertial properties and locomotor ability

#### Sensorimotor control

Isometric and positively allometric scaling of limb inertial properties may not offer an energetic benefit towards terrestrial locomotion, but how does the scaling of limb inertial properties relate to other aspects of terrestrial locomotion? From a sensorimotor perspective, large values of limb inertia in either absolute or relative terms may not necessarily pose a severe limitation upon the locomotor system. Within terrestrial mammals, axonal conduction velocity is nearly independent of body mass, and axon diameter only increases roughly by a factor of two between a 5 g shrew (*Cryptotis parva*) and a 3,680 kg elephant (*Elephas maximus*) [70]. Thus, larger mammals have a greatly delayed sensorimotor response time compared to smaller mammals. Consequently, the delayed response times of large-bodied mammals may drastically limit rapid movements of their limbs, regardless of their greater values of limb inertia.

#### Locomotor potential

Similarly, the scaling of limb inertial properties with increasing body mass may be intimately tied to safety factors and reduced locomotor abilities of larger bodied mammals. To maintain similar safety factors between smaller and larger bodied mammals, larger mammals appear to have a reduced locomotor potential or agility so as to reduce peak bone stresses [71]–[73]. Moreover, larger bodied mammals also have lower maximal running speeds than some mammals of lower body mass [74]. If slower limb movements are necessary for reducing peak locomotory stresses and maintaining adequate safety factors in larger mammals, then the greater limb inertia of the larger mammals may coincide with otherwise existing limits on locomotor potential in these larger taxa. It is also plausible that a greater relative concentration of mass in the distal limb segments may impart greater stability during locomotion to larger bodied mammals.

#### Kinematics

According to geometric similarity, ‘physiologically equivalent’ speeds, such as gait transition speeds and preferred speeds within a gait, are predicted to scale independently of body mass [13], [75]. To the contrary, physiologically equivalent speeds are positively allometric with body mass, scaling approximately as M^0.21^. It thus appears that isometry and positive allometry of limb inertia does not limit the preferred speeds and gait transitions used by larger bodied mammals. However, stride frequency at these physiologically equivalent speeds may be only mildly adversely affected by limb inertia, as it decreases as approximately M^−0.14^ [18], which is significantly greater than geometric similarity's prediction of M^-1/3^ [13]. Indeed, fore- and hindlimb natural frequency scale as M^−0.20^ and M^−0.18^, respectively, suggesting that the scale effects within mammalian limb morphology do not limit the scaling of stride frequency. In spite of the positive allometric and isometric scaling of limb inertia, the positive allometry of stride frequency may be possible due to anatomical specializations aiding in limb protraction or retraction via the storage of elastic strain energy. While such specializations have been suggested or identified in isolated species [76]–[78], it remains unknown how widespread and effective such specializations are across terrestrial mammals and how such specializations vary with body size. Nonetheless, the scaling of stride frequency with body mass in all likelihood partly enables the positive allometry of preferred and gait transitions speeds, as do the positive allometry of stride length [67] and limb length (current study).

An additional factor that would influence the scaling of limb inertial properties, in addition to their effects on locomotor potential, is the degree of limb flexion that occurs during the swing phase of locomotion. Increased flexion of the limb would bring the mass of the distal limb segments closer to the limb's pivot, and reduce the limb's COM distance and radius of gyration. This, in turn, would have the overall effect of reducing the limb's MOI. In spite of known postural differences between smaller and larger mammals [26], it remains unclear how limb flexion during swing phase scales with increasing body size in terrestrial mammals. The *a priori* prediction would be that larger bodied mammals exhibit greater limb flexion than smaller bodied mammals, so as to offset the greater MOI of their limbs in both absolute and relative terms. Interestingly, this would be counter to known postural differences regarding the role of limbs in supporting body mass, with larger mammals having less flexed limbs, and smaller mammals having more crouched limbs [25]–[26]. Such size dependent changes in limb flexion might also relate to how stride frequency scales in mammals. However, rigorously examining the influence of size and speed upon the joint flexion during swing phase exceeds the limits and scope of the current study.

Yet previous studies do indicate that limb inertia does influence mammalian quadrupedal locomotion. To examine the influence of limb inertia, previous studies have attached leg weights to the distal limb segments of dogs (*Canis familiaris* [9]–[10]) and horses (*Equus caballus* [12]). In both these taxa, adding weight to the distal limb segments resulted in an increase in metabolic locomotor costs. In the horse, adding weight to the distal limb segments also resulted in increased flexion of the hindlimb, which would reduce limb rotational inertia, though forelimb flexion showed no increase. This differential response in limb flexion between fore- and hindlimbs has also been documented in dogs [79] and is likely owed to differences between fore- and hindlimbs regarding the ability of extrinsic and intrinsic limb muscles to produce force and mechanical power.

It should also be noted that terrestrially locomoting mammals, including didelphimorphs [80], rodents [81]–[82], perissodactyls [83]–[84], artiodactyls [85], carnivorans [86]–[87], primates [88]–[89], and proboscideans [90], tend to increase speed by primarily decreasing the duration of stance phase. In contrast, the duration of swing phase remains constant with respect to velocity or only exhibits a minor decrease at low velocities before becoming largely invariant. If limb inertia imposes a limit on how quickly limbs can be accelerated and decelerated, then this might explain the largely invariant swing phase duration documented in several species of terrestrially locomoting mammals [82].

#### Foot impacts

While limb inertia is obviously relevant for swing phase, limb inertia is also relevant to the impact of the limb with the ground during the start of stance phase, prior to the limb being loaded with body mass. In a study of impact force scaling in ungulates, Warner et al. [91] found that the impact forces scale either with negative allometry or isometry that trends towards negative allometry. Our finding of isometric and positively allometric scaling of limb mass and MOI may limit scale effects in foot impact force to isometry or negative allometry, given that the exponents for mass and MOI are greater than the exponent for scaling of muscle force (M^0.80^) [92]. However, before definitive statements can be made between the results of our study and those of Warner et al. [91], some caveats must be stressed. In our study, we did not measure the inertia of individual limb segments, in particular the manus or pes. While Warner and colleagues did measure velocity of foot impact, they did not measure acceleration of foot impact, which is of vital importance in relating forces to inertia. With data on segment inertia and impact accelerations, as well as similarity in taxonomic sampling, the relationship between scale effects in limb morphology and impact dynamics can be better understood, a topic highly worthy for future study.

### Angular acceleration

As MOI is resistance to angular acceleration, the scaling of limb MOI with body mass can provide insight as to how limb angular acceleration scales with body mass. The angular acceleration (α) exerted about a limb is the quotient of the sum of the moments generated by muscles acting about the limb's pivot and limb MOI:
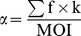
(5)


with *f* being the force exerted by an individual muscle acting across the limb's pivot and *k* being the corresponding moment arm of the muscle.

Geometric similarity predicts that limb MOI should scale as M^5/3^, whereas the maximum force that muscles can exert should scale as M^2/3^. According to geometric similarity, muscle moment arms should scale as M^1/3^. If we assume that muscle moment arms scale isometrically with body mass (M^1/3^), the maximum angular acceleration that can be applied to the limbs should scale according to the following relationship:

Predicted Exponent:
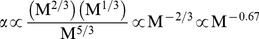
(6)


Thus following geometric similarity, as body mass increases the potential for limb angular acceleration decreases relative to body mass.

With regards to hindlimbs, limb MOI scales according to geometric similarity; however, the peak force exerted by the extrinsic limb muscles scales with positive allometry. Peak muscle force scales as M^0.80^ [92], as opposed to M^2/3^ predicted by geometric similarity. While no equivalent data exist for hindlimb proximal muscles, the moment arms of ankle extensors scale as M^0.38^ [92]. Noting that f ∝ M^0.80^, hindlimb MOI ∝ M^1.66^, and k ∝ M^0.38^ (assuming that all hindlimb moment arms scale in a similar way with body mass), Equation 5 can be rewritten as the following proportionality:

Derived Exponent:
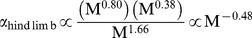
(7)Equation 7 illustrates that although the ability to impart angular acceleration to the hindlimbs still declines with increasing body size, larger mammals have a greater potential to angularly accelerate the hindlimb than would be expected from geometric similarity. This conclusion still appears to hold true when using confidence limits on scaling exponents for muscle force, moment arms, and hindlimb MOI, though the lower confidence limit borders on the prediction of geometric similarity (Table 10).

**Table 10:** Confidence limits on the scaling exponent for angular accelation potential of both fore- and hindlimbs across terrestrial quadrupedal mammals.

	Muscle Force	Moment Arm	MOI	Limit
**Hindlimb**				
Upper	M^0.85^	M^0.41^	M^1.56^	M^−0.30^
Lower	M^0.75^	M^0.35^	M^1.76^	M^−0.66^
**Forelimb**				
Upper	M^0.86^	M^0.44^	M^1.68^	M^−0.39^
Lower	M^0.75^	M^0.38^	M^1.89^	M^−0.76^

Upper confidence limits for angular acceleration potential were generated by using the upper confidence limits for muscle force and moment arms and lower confidence limits on limb MOI in order to maximize angular acceleration potential (see Eq. 5). Likewise, lower confidence limits for angular acceleration potential were generated by using lower confidence limits for muscle force and moment arms and upper confidence limits on limb MOI. Confidence limits on muscle force and muscle moment arms were obtained from Alexander et al. [91], while confidence limits on limb MOI are from the current study.

With regards to the forelimbs, limb MOI scales as body mass raised to a power of 1.79, and the moment arm of the triceps scales as body mass raised to a power of 0.41 [92]. Thus using the proportionalities f ∝ M^0.80^, MOI ∝ M^1.79^, k ∝ M^0.41^ (assuming that all forelimb moment arms scale similarly with body mass), an allometric exponent describing how forelimb angular acceleration should scale with body mass can be derived:

Derived Exponent:
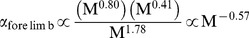
(8)


As is the case with the hindlimb, forelimb angular acceleration is greater in larger mammals than is predicted from geometric similarity. However, when using scaling exponents for muscle force, moment arms, and forelimb MOI, confidence limits for forelimb angular acceleration potential include the null model prediction (Table 10). It should be noted though that the above exponents in Equations 7 and 8 and Table 10 could be underestimates. If the flexion of the limbs during swing phase increases with both body and limb size, then limb MOI should have a lower exponent, which would result in greater exponents for angular acceleration potential.

### Comparisons to previous studies

Previous studies have found that both limb muscle and long bone mass scale with positive allometry or isometry with respect to body mass. Alexander et al. [92] found that the mass of adductors, quadriceps, and triceps each scale via positive allometry, whereas the mass of wrist flexors and hind flexors scale isometrically. Alexander and colleagues' result of positive allometry of muscle mass appears to be at odds with our result of isometry of limb mass. However, the study of Alexander and colleagues was not inclusive of all the muscles in the fore- and hindlimb. Within their study, adductors referred to the *adductor magnus, biceps femoris, semitendinosus, semimembranosus*, and *gracilis*, whereas quadriceps referred to the *rectus femoris* and the *vastus*. Consequently, it is unknown how the mass of the remaining limb muscles, such as the *pectoralis* and gluteal muscles, *tensor fasciae latae*, and *sartorius*, scale with body mass. In light of this, it is plausible that the total muscle mass of fore- and hindlimbs scales isometrically with body mass, as muscles apart from the triceps, adductors, and quadriceps could scale with negative allometry or isometry.

Christiansen [93] reported that the masses of forelimb long bones scale with positive allometry relative to body mass, while the masses of hindlimb long bones scale with isometry. In our study, we found that the mass of both fore- and hindlimbs scales isometrically with body mass. One possible reason for this contrast in results is differences in sampling between our study and Christiansen's. Only 11 taxa are shared between the current study (N = 44) and the work of Christiansen (N = 64). While our study and Christiansen's sample several of the same orders, including Artiodactyla, Perissodactyla, Hyracoidea, and Carnivora, our study additionally samples Rodentia, Lagomorpha, Cingulata, and Didelphimorphia. Christiansen's study sampled Proboscidea, as well as graviportal artiodactyls and perissodactyls, which we regrettably could not sample for our study. Given the different scaling trends in mammalian subclades (Table 9), these differences in subclade sampling could underlie the differing results between our study and Christiansen's. Additionally, an order of magnitude difference in body mass existed between the smallest taxa in our and Christiansen's studies, as well as an order of magnitude difference in body mass between the largest taxa in our two studies. If differential scaling occurs in the scaling of long bone mass or limb mass between small and large mammals [94], then including or excluding taxa at either extreme of body size could have a strong influence upon overall scaling trends for Mammalia.

## Conclusions

The scaling of limb length has a strong potential to underlie COT scaling in quadrupedal mammals, as the positive allometry of limb length likely allows for larger mammals to take relatively longer strides and to utilize a fewer number of strides to travel a given distance. However, limb inertial properties do not have the potential to underlie COT scaling. Across quadrupedal mammals, limb mass scales isometrically with body mass. For the remaining inertial properties, fore- and hindlimbs scale differently. Forelimb MOI, COM position, and radius of gyration all scale with positive allometry. In contrast, in the hindlimb, all these traits scale isometrically. Within mammalian subgroups, limb length and inertial properties tend to scale according to geometric similarity, though exceptions occur within cursorial mammals as a group. All limb length and inertial properties individually possess strong phylogenetic signal; however, only the residuals of regressions between limb length and body mass carry significant phylogenetic signal. This suggests that shared ancestry has strongly influenced the coevolution of body mass and limb length, while phylogeny does not appear to strongly influence how body mass had evolved alongside limb inertial properties. The results of phylogenetic regressions differ from conventional regressions, likely due to differences in scaling trends and absolute limb design among mammalian subgroups. While isometric and positively allometric scaling would appear to have a detrimental effect upon the cost of swinging the limbs, the scaling of limb inertial properties coincide with how sensorimotor control and locomotor ability scale with body mass in terrestrial mammals. Moreover, the scaling of limb inertial properties do not seem to limit the preferred speeds and stride frequencies chosen by larger bodied mammals. Yet to know the exact significance of limb rotational inertia in mammalian locomotion, futures studies must examine how small- and large-bodied mammalia differ in limb flexion during terrestrial locomotion. We hope that the results of our current study can give more impetus into examining how differences in overall limb morphology contribute to locomotor specializations.

## Supporting Information

File S1 **Supporting file providing detailed methodology for dissection and inertial property measurement (Table S1 and Figure S1), derivation of scaling relationships, tree construction methodology and comparative methods results (Tables S2 to S5), and appendix of inertial property values (Tables SA1 and SA2).**
(PDF)
