# Supplementary material for: Scale Effects between Body Size and Limb Design in Quadrupedal Mammals
Source: PLoS One. 2013 Nov 8;8(11):e78392. doi: 10.1371/journal.pone.0078392 (PMC3832634; doi:10.1371/journal.pone.0078392)
Supplement: File S1 — Supporting file providing detailed methodology for dissection and inertial property measurement (Table S1 and Figure S1), derivation of scaling relationships, tree construction methodology and comparative methods results (Tables S2 to S5), and appendix of inertial property values (Tables SA1 and SA2). (PDF) [file pone.0078392.s001.pdf]

SUPPLEMENTARY MATERIALS

MATERIALS & METHODS

Carcass quality

Sampled specimens were either 1) salvaged specimens from the wild donated to the Field Museum of Natural History and the Iziko South African Museum by zoos and local, state/provincial, or national wildlife agencies or 2) freshly culled individuals from federal and state wildlife agencies, the University of Stellenbosch, and South African game farms. Specimens donated to the two museums were stored in deep freezers in sealed plastic bags. After thawing and prior to dissection, specimens were thoroughly inspected for any signs of freeze drying and consequential loss of mass and/or joint mobility. Specimens exhibiting significantly levels of free drying were excluded form out study. The only exception to this was for specimens with freeze dried interphalangeal joints. For these specimens, the manus and pedes were wrapped in water soaked bandages 12 to 24 hours prior to dissection. The bandages were secured with rubber bands and water was added to re-soak the bandages regular intervals of 1 to 2 hours. Furthermore, none of the specimens studied exhibited any visible signs of decomposition or putrefaction. In the case of the Field Museum of Natural History, all specimens were required to have tissue samples taken for molecular sequencing; as such, the condition of the studied specimens had to further meet this requirement. Additionally, none of the thawed specimens exhibited any signs of rigor mortis.

With regards to freshly culled mammals, limbs were dissected from carcasses within one to two hours of death, at which time they had their full range of joint mobility. After dissecting the limbs from the carcass, great care was taken to place the limbs in their ‘passively flexed length’ (see below) prior to rigor mortis setting in. While slipping into rigor, the limbs did not arbitrarily flex from the set position; however, once rigor set in, the joints of the limbs could not be flexed or extended.

Prior to data collection, specimens at the Field Museum of Natural History and Iziko South African Museum were determined to be adult individuals by visual inspection of body size and initial examination of tooth wear. Upon completion of data collection, these specimens were skeletally prepared by B.M.K. After skeletal preparation, specimens were further examined more closely for tooth wear and additionally for fusing of epiphyseal sutures to make certain that individuals were adults. In the case of specimens obtained from game farms, specimens were determined to be adult individuals through visual inspection of their body size, assessment of their body mass, the development of horns, and consultation with game farm staff.

Limb Dissection

Limbs were removed preferably with the skin still *in situ* on the limb. However, in some cases the specimen was skinned prior to dissection to accommodate needs of colleagues that donated the specimen to this study. Since we dissected limbs from the carcass with skin overlying the limb muscles, we used osteological markers to position

the incisions necessary to remove the limb. Forelimbs were removed by making a sagittal incision along the length of the sternum, followed by a posterior incision from the caudal angle of the scapula to the sternal incision. The posterior incision was just caudal to the axilla of the forelimb so as to include the whole of the forelimb. We next made an incision from the caudal angle of the scapula along the dorsal and cranial margins of the scapula to the glenoid joint and a final incision from the glenoid joint to the cranial end of the manubrium. To free the limb from the torso, we dissected the pectoralis muscle group from the body wall and shaved the serratus ventralis from the medial surface of the scapula. For species possessing clavicles, the clavicle was included as part of the forelimb by severing the ligaments between the clavicle and the manubrium.

We removed hindlimbs by making a vertical incision just cranial to the crest of the ilium. A second incision was made starting at the previous incision and the dorsal border of the ilium and continuing to the caudal-most extent of the ischiadic tuberosity, parallel to the neural spines of the sacral vertebrae. These incisions exposed the anterior and dorsal edge of ilium and ischium. We then proceeded to shave the limb muscles off the lateral surface of the ilium, cutting through the joint capsule of the hip and the ligament of the femoral head. After shaving the limb muscles off the ilium and freeing the femoral head from the acetabulum, we then shaved along the ventrolateral face of the pelvis down to pelvic symphysis, freeing the whole limb from the pelvis.

Measuring inertial properties

All limbs were fully thawed. After first weighing the limb, its length was measured. The length was taken after fully extending the limb and then releasing it to flex on its own accord. The distance from the limb’s pivot to distal-most extreme of the limb was then taken as the limb’s length. In the case of the hindlimb, the limb’s pivot is the femoral head. For the entire forelimb (i.e., the scapula and all segments distal) the pivot was considered to be at the intersection of the scapular spine and the vertebral margin of the scapula [S1-S3]. For the forelimb with the scapula removed, the humeral head was considered the limb’s pivot. The passively flexed length of the limb was taken as the

We next attached the limb to a bar with known inertial properties (mass, center of mass position, moment of inertia, radius of gyration, and natural frequency) in the extended (i.e., straightened) position using cable ties. Limbs were attached to this bar 1) to prevent flexion of the limb during data collection and 2) to be able to mount the limb onto a pivot consisting of a rotary shaft passing through two bearings. A shaft clamp on the rear face of the bar coupled the bar and its attached limb to the shaft of the pivot. When attaching the limb to the bar, the limb’s pivot point during locomotion was aligned with the shaft clamp, insuring that inertial properties were measured with respect to the limb’s axis of rotation during locomotion. As the mass of the limbs varies greatly across species, several bars of differing length, mass, and moment of inertia were used for data collection to minimize differences between inertial properties of the bar and those of the limb.

To find limb COM position, we suspended the limb-bar combination from two spring scales. Using a tape measure, we then measured the distance from the limb's pivot to each spring scale. We calculated the COM position of the combined limb and bar using the following equation which balances the moments acting on the bar and limb:

$$COM_{total} = \frac{(s_1 d_1) + (s_2 d_2)}{m_{total}} \tag{S1}$$

where COM_{total} equals the COM position of the combined bar and limb and m_{total} equals the total mass of both the bar and limb. For the spring scale proximal to the limb's pivot, s_1 and d_1 respectively correspond to the scale's reading and its distance to the limb's pivot. s_2 and d_2 likewise correspond to the second scale's reading and its distance to limb's pivot. Knowing the inertial properties of the bar, we used the following equation to find the limb's COM position:

$$COM_{limb} = \frac{[(m_{total} COM_{total}) - (m_{bar} COM_{bar})]}{m_{limb}} \tag{S2}$$

where COM_{limb} is the limb's COM position, COM_{bar} is the bar's COM position, m_{limb} is limb mass, and m_{bar} is bar mass.

After finding limb COM position, we proceeded to determine the moment of inertia and natural frequency of the limb. Mounting the combined limb and bar onto a pivot via the bar's shaft clamp, we deflected the limb and attached bar from the their rest position by a small angle (~20-30°) and then released them to swing freely in a parasagittal plane. While the limb was swinging, we videotaped it at 30 fps for 15 cycles. For taxa with smaller limbs (e.g., *Sciuru*s, *Tamias*, etc), we videotaped limbs at 90-120 fps. Using the video, we determined the natural period (the inverse of natural frequency) of the limb and bar, treating them as a single object. We were then able to calculate the moment of inertia of the limb by using the following equation from Myers & Steudel (1997):

$$MOI_{limb} = [(m_{limb} COM_{limb}) + (m_{bar} COM_{bar})] g (t_{total} / 2\pi)^2 - MOI_{bar} \tag{S3}$$

where MOI_{limb} is the moment of inertia for a limb, g is gravitational acceleration (= 981 cm/s^2), t_{total} is the natural period of the bar, the limb, and the shaft of the pivot as one unit, and MOI_{bar} is the moment of inertia of the bar attached to the shaft. In Equation S3, the variables m_{bar} and COM_{bar}, refer to the bar fixed to the shaft; m_{bar} and COM_{bar} were recalculated to account for the shaft's mass and its effect upon the bar's COM position, as the bar is securely attached to the shaft as it swings. In Equation S3, m_{bar} is the total mass of the bar and the shaft, and COM_{bar} is the COM position of the bar after the shaft has been secured to the bar using the shaft clamp.

Now knowing the moment of inertia of the limb, I proceeded to calculate limb natural frequency. we calculated limb natural frequency as follows:

$$F_{limb} = \frac{1}{2\pi} \sqrt{\frac{m_{limb} COM_{limb} g}{MOI_{limb}}} \tag{S4}$$

with F_{limb} being limb natural frequency. Also, using Equation 1, we determined limb radius of gyration by using the mass and moment of inertia of the limb. Species means for limb inertial properties of both fore- and hindlimbs are given in the Appendix A.

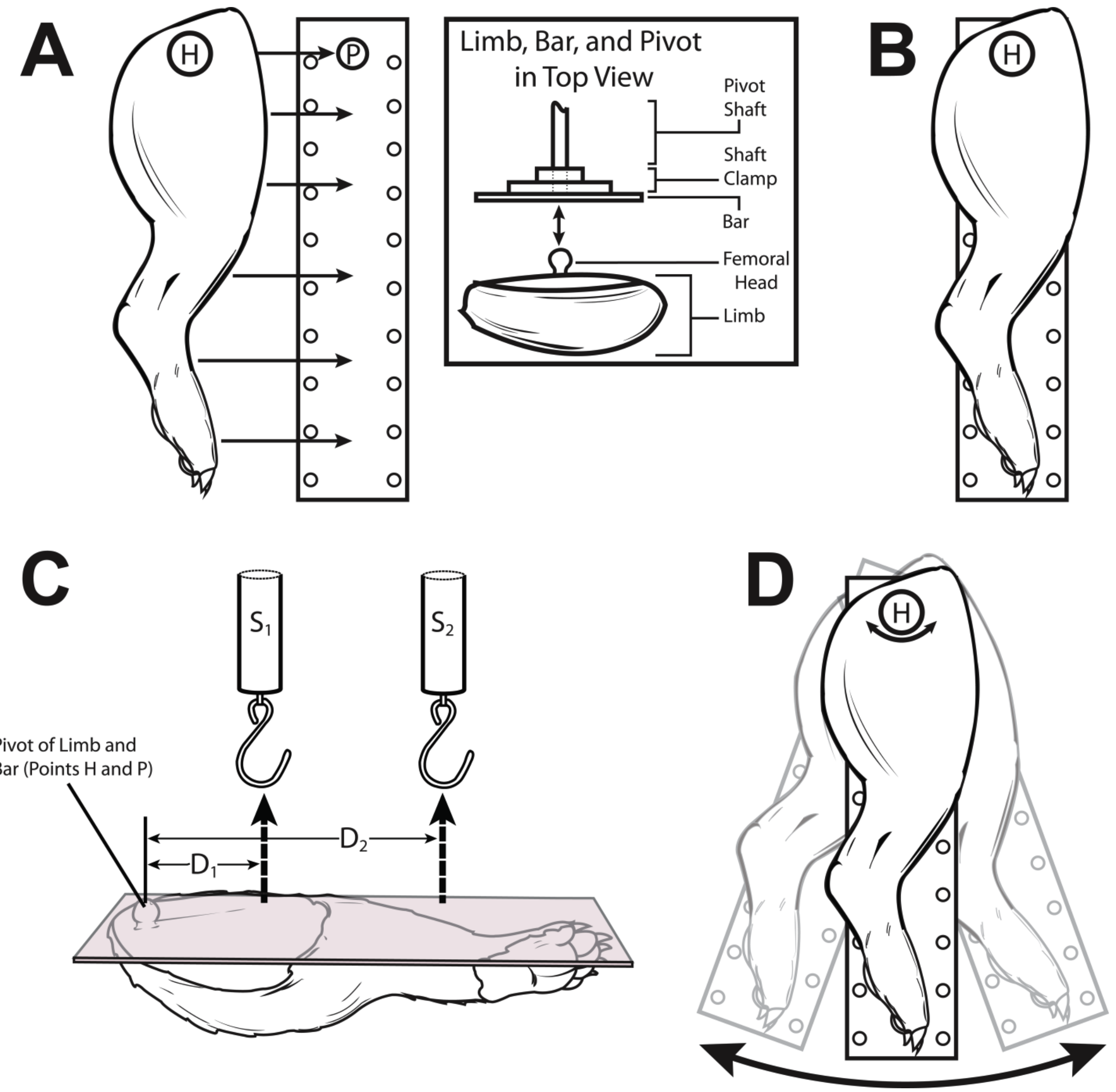

Figure S1. Experimental setup to measure limb inertial properties. After dissecting the limb from the torso and weighing it, the limb is attached in its passively flexed position to a bar with known inertial properties, with the pivot of the limb (Point H) overlying the pivot of the bar (Point P) (A and inset). The position of the limb on the bar is shown in B. Next the combined limb and bar are suspended from two spring scales that are a known distance from the pivot of the limb and bar (C). Knowing the distance of each scale from the pivot (D_1 and D_2), the readings of the scale (S_1 and S_2), and the mass of the limb and bar, the limbs's COM position is determined using Equation S1 and then Equation S2. Next, the combined limb and bar are set upon a pivot (inset), and the limb is allowed to freely swing back and forth on the pivot while being videotaped (D). Knowing the number of frames it takes for the combined limb and bar to complete a full oscillation and the frame rate, the time required for the combined limb and bar to complete a full oscillation can be calculated (i.e., its natural period). Using Equations 1 (main text), S3, and S4, we then calculated the limb's MOI, radius of gyration, and natural frequency.

Sources of methodological error

We should address two potential sources of error lie in our methodology. The first source is our identification of the scapula's point of rotation. The scapula's contribution to forelimb kinematics is undeniable [S4]; however, the exact location of the pivot during both stance and swing phases remains unknown for the bulk of species sampled in our study, as well how the pivot's position varies with size across species. Previous studies have found that the scapular pivot lies within the proximal third of the scapula where the scapular spine crosses the vertebral margin of the scapula [S1-S2]. We therefore measure forelimb inertial properties with respect to the intersection of the scapular spine and scapula's dorsal edge. By measuring forelimb COM position, MOI, radius of gyration, and natural frequency all with respect to the same anatomical landmark for all individuals, the data can be corrected as more detailed information on scapular kinematics becomes available for a greater diversity of species.

The second potential source for error is the extent to which the limb is flexed/extended while collecting data. As limbs were not frozen at the time of study, minor amounts of joint flexion may have been added inadvertently when mounting the limb for study. To test the effect that different degrees of limb flexion may have had upon the measurement of limb inertial properties, limb inertial properties were measured for a single hindlimb of a grey wolf (*Canis lupus*) over five separate trials (Table S1). The coefficient of variation is less than 5.0% for all the variables measured over these five trials. Such low values for the coefficient of variation indicate that the data are only weakly affected by differences in limb flexion between studied limbs.

Trial	Mass	Length	COM	Relative COM	MOI	Radius of Gyration	Relative Radius of Gyration	Natural Frequency
	(g)	(cm)	(cm)	(%)	($g*cm^2$)	(cm)	(%)	Hz
01	2348	61.4	14.4	23.5	849635.4	19.0	31.0	1.00
02	2316	61.4	14.2	23.2	802060.9	18.6	30.3	1.01
03	2312	61.2	13.9	22.7	806902.3	18.7	30.5	1.00
04	2312	61.3	13.6	22.2	770863.5	18.3	29.8	1.01
05	2306	61.3	14.1	23.1	850035.4	19.2	31.3	0.98
Mean	2318.8	61.3	14.0	22.9	815899.5	18.8	30.6	1
St. Dev.	16.71	0.083	0.305	0.503	33926.6	0.351	0.589	0.012
CV (%)	0.7	0.1	2.2	2.2	4.2	1.7	1.9	1.2

Table S1. Estimation of measurement error due to differences in limb flexion between measured limbs. Limb inertial properties were measured for a single hindlimb of *Canis lupus* over five separate trials. CV refers to the coefficient of variation.

Null model predictions

Central to geometric similarity are explicit relationships between length and diameter. According to the model [S5-S6], linear dimensions remain in direct proportions with one another as body size increases. Providing us with the relationship:

$$l \propto d^{1.0} \tag{S5}$$

where *l* and *d* are length and diameter, respectively. Therefore to increase the size of an organism by a factor of 2, all linear dimensions would only have to be multiplied by a factor of 2. This would be the case for any factor *n*.

Mass and length. Of primary interest for the scaling of limb inertial properties is how limb length relates to mass, regardless of whether it is body mass (M) or limb mass (m) under examination. Mass is the product of density (ρ) and volume (V) and volume is a product of length and area ($\pi(d/2)^2$), we can write the following equation:

$$M = \rho V = \rho\pi(d/2)^2 l \tag{S6}$$

and from Equation S6 we can write the following proportionality:

$$M \propto ld^2 \tag{S7}$$

Given proportionalities S5 and S7, the following proportionality can be written between length and mass for geometric similarity:

$$l \propto M^{1/3} \tag{S8}$$

Mass distribution and MOI. Center of mass (COM) position was taken as the length from the limb's COM to the limb's axis of rotation, and the radius of gyration (r) is the length between the centroid of the moment of inertia (MOI) and the limb's axis of rotation. Thus COM position and radius of gyration scale with mass according to the following proportionalities for the null model:

$$COM \propto M^{1/3} \tag{S9}$$

$$r \propto M^{1/3} \tag{S10}$$

MOI is a product of limb mass and the square of the radius of gyration: MOI = $m \cdot r^2$. It is important here to note that geometric similarity also predicts that changes in mass are also directly proportional, such that $M \propto m$ [S7-S8]. By using proportionality S10, the following relationships between limb MOI and mass can be derived:

$$MOI \propto mr^2 \propto M^{1.0}\left(M^{1/3}\right)^2 \propto M^{1.67} \tag{S11}$$

*Natural frequency***.** The formula for calculating the natural frequency of a gravitational pendulum is:

$$F = 2\pi\sqrt{\frac{COM(m)g}{MOI}} \tag{S12}$$

where F is the natural frequency and g is the gravitational constant. From S11 and S12, the following proportionality can be written:

$$F \propto \sqrt{\frac{COM(m)}{mr^2}} \tag{S13}$$

The mass terms cancel out in S13, and, recognizing that COM $\propto$ r from Equations S9 and S10, proportionality S14 is produced:

$$F \propto r^{-0.5} \quad \text{(S14)}$$

By substituting the relationships for the radius of gyration to mass from S10 into S14, we can predict how limb natural frequency scales to body mass under geometrically similar scaling.

$$F \propto \left(M^{1/3}\right)^{-0.5} \propto M^{-1/6} \quad \text{(S15)}$$

Comparative Methods

The taxa included in this study span eight taxonomic orders. In order to perform comparative methods, a composite phylogeny had to be constructed (Fig. 1). Relationships between mammalian orders and families were based upon Meredith et al [S9]. Topologies for mammalian orders and families were based off published phylogenies for artiodactyls [S10-S12], carnivorans [S13-S16], rodents [S17-S18], and perissodactyls [S19]. In order to scale branch lengths to divergence time across these different phylogenies, divergence time branch lengths for all phylogenies cited were scaled to the divergence times of Meredith et al. [S9] by means of common, dated nodes shared by Meredith et al. [S9] and the remaining phylogenies.

**Node**	**Date**	**Primary Source**	**Secondary Source**	**Tertiary Source**
Theria	189.85	[S9]		
Placentalia	101.35	[S9]		
Exafroplacentalia	99.38	[S9]		
Boreoeutheria	92	[S9]		
Fereungulata	81.98	[S9]		
Euungulata	80.70	[S9]		
Carnivora	54.67	[S9]		
Caniformia	46.49	[S9]		
Canidae	12.38	[S16]	[S9]	
Arctoidea-Pinnepedia	30.42	[S15]	[S9]	
Mustelidae+Procyonidae	26.05	[S15]	[S9]	
Mustelidae	12.45	[S15]	[S9]	
Mustelidae-Taxidea	8.17	[S14]	[S15]	[S9]
Felidae	9.51	[S15]	[S9]	
Felidae (Internal Nodes)		[S13]	[S15]	[S9]
Glires	79.5	[S9]		
Rodentia	68.96	[S9]		
Muridae+Cricetidae	25.88	[S9]		
Hystricognathi	49.03	[S9]		
Castorimorpha+Myomorpha	65.1	[S9]		
Sciuromorpha+Hystricomorpha	67.36	[S9]		
Sciuridae	36.25	[S18]	[S9]	
Sciuridae (Internal Nodes)		[S17]	[S18]	[S9]
Hippomorpha	3.8	[S18]	[S9]	
Ruminantia-Tragulidae	20.4	[S9]		
Bovidae+Cervidae	19.63	[S9]		
Bovidae (and Internal Nodes)	15.58	[S11]	[S12]	[S9]
Cervidae (and Internal Nodes)	11.92	[S10]	[S9]	

Table S2. Phylogenies used to scale divergence times within the composite phylogeny. Node names refer to those referenced in Meredith et al. [S9].

Results of tests for phylogenetic signal

**Trait**	**λ**	**λ C. L.**	**$\lambda_{Residuals}$**	**$\lambda_{Residuals}$ C. L.**
Body Mass	0.91	0.725, 0.992	--	--
		Forelimbs		
Length	0.94	0.794, 0.996	0.87	0.410, 0.965
Mass	0.90	0.598, 0.967	< 0.0001	< 0.0001, 0.142
COM Position	0.91	0.606, 0.982	0.54	< 0.0001, 0.749
MOI	0.91	0.590, 0.983	0.31	< 0.0001, 0.591
Radius of Gyration	0.92	0.638, 0.985	0.65	< 0.0001, 0.828
Natural Frequency	0.93	0.743, 1.000	0.73	< 0.0001, 0.889
		Hindlimbs		
Length	0.94	0.616, 0.993	0.89	0.635, 0.970
Mass	0.92	0.744, 0.990	< 0.0001	< 0.0001, 0.487
COM Position	0.87	0.263, 0.951	0.52	< 0.0001, 0.756
MOI	0.92	0.594, 0.988	< 0.0001	< 0.0001, 0.009
Radius of Gyration	0.91	0.708, 0.989	0.56	< 0.0001, 0.856
Natural Frequency	0.92	0.792, 0.980	0.53	< 0.0001, 0.785

Table S3. Phylogenetic signal within individual traits and within residuals of body mass regressions when branch lengths are scaled to divergence time. ‘C. L.’ denotes 95% confidence intervals.

Trait	λ	λ C. L.	$\lambda_{Residuals}$	$\lambda_{Residuals}$ C. L.
Body Mass	0.92	0.681, 0.980	--	--
		Forelimbs		
Length	0.97	0.759, 1.000	0.99	0.875, 1.000
Mass	0.91	0.605, 1.000	0.38	< 0.0001, 0.745
COM Position	0.92	0.640, 1.000	0.80	0.238, 0.972
MOI	0.92	0.493, 1.000	0.41	< 0.0001, 0.736
Radius of Gyration	0.93	0.540, 1.000	0.84	0.339, 0.979
Natural Frequency	0.94	0.452, 1.000	0.88	0.561, 1.000
		Hindlimbs		
Length	0.97	0.634, 1.000	0.99	0.665, 1.000
Mass	0.93	0.201, 1.000	0.53	< 0.0001, 0.871
COM Position	0.93	0.742, 1.000	0.86	0.422, 0.982
MOI	0.93	0.216, 1.000	0.34	< 0.0001, 0.751
Radius of Gyration	0.93	0.598, 1.000	0.80	0.305, 0.976
Natural Frequency	0.93	0.637, 1.000	0.50	< 0.0001, 0.753

Table S4. Results of PGLS regressions for Mammalia when using branch lengths scaled to unity. ‘C. L.’ denotes 95% confidence intervals.

Trait	λ	λ C. L.	Intercept	Slope	Slope C. L.	L	*P*
			Geologic Age				
Fore Length	0.90	0.799, 1.010	0.20	0.33	0.297, 0.370	52.7	< 0.0001
Hind Length	0.93	0.839, 1.018	0.31	0.30	0.266, 0.337	53.2	< 0.0001
			Unity				
Fore Length	0.90	0.798, 1.010	0.20	0.33	0.297, 0.370	52.7	< 0.0001
Fore COM Position	0.62	0.116, 1.118	-0.24	0.33	0.290, 0.373	44.1	< 0.0001
Fore Rad. Gyration	0.74	0.344, 1.130	-0.21	0.34	0.299, 0.382	45.3	< 0.0001
Hind Length	0.93	0.839, 1.018	0.31	0.30	0.266, 0.337	53.2	< 0.0001
Hind COM Position	0.62	0.143, 1.088	-0.17	0.27	0.220, 0.323	34.7	< 0.0001
Hind Rad. Gyration	0.65	0.203, 1.090	-0.09	0.29	0.247, 0.332	43.6	< 0.0001

Table S5. Results of PGLS regressions that simultaneously estimate λ along with regression coefficients [S20]. ‘C. L.’ denotes 95% confidence intervals.

SUPPORTING REFERENCES

S1. Boczek-Funcke A, Kuntz-Buschbeck JP, Illert M (1996) Kinematic analysis of the cat shoulder girdle during treadmill locomotion: an x-ray study. Eur J Neurosci 8: 261-272.

S2. Schmidt M, Fischer MS (2000) Cineradiographic study of forelimb movements during quadrupedal walking in the brown lemur (*Eulemur fulvus*, Primates: Lemuridae). Am J Phys Anthro 111: 245-262.

S3. Fischer MS, Schilling N, Schmidt M, Haarhaus D, Witte H (2002) Basic limb kinematics of small therian mammals. J Exp Biol 205: 1315-1338.

S4. Fischer MS, Blickhan R (2006) The tri-segmented limbs of therian mammals: kinematics, dynamics, and self-stabilization—a review. J Exp Zool 305A: 935–952.

S5. Hill AV (1950) The dimensions of animals and their muscular dynamics. Sci Prog 38: 209-230.

S6. McMahon TA (1975) Using body size to understand the structural design of animals: quadrupedal locomotion. J App Physiol 39: 619-627.

S7. McMahon TA (1984) *Muscles, Reflexes, and Locomotion*. Princeton, USA: Princeton University Press.

S8. Prothero J. 1992. Scaling bodily proportions in adult terrestrial mammals. Am J Physiol Regulatory Integrative Comp Physiol 262: 492-503.

S9. Meredith RW, Janecka JE, Gatesy J, Ryder OA, Fisher CA, et al. (2011) Impacts of Cretaceous terrestrial revolution and KPg extinction on mammalian diversification. Science 334: 521-524.

S10. Fernández MH, Vrba ES (2005) A complete estimate of the phylogenetic relationships in Ruminantia: a dated species-level supertree of the extant ruminants. Biol Rev 80: 269-302.

S11. Robinson TJ, Ropiquet A (2012) Examination of hemiplasy, homoplasy, and phylogenetic discordance in chromosomal evolution of the Bovidae. Syst Biol 60: 439-450.

S12. Hassanin A, Delsuc F, Ropiquet A, Hammer C, van Vuuren BJ, et al. (2012) Pattern and timing of diversification of Cetartiodactyla (Mammalia, Laurasiatheria), as revealed by comprehensive analysis of mitochondrial genomes. C R Biologies 335: 32-50.

S13. Johnson WE, Eizirik E, Pecon-Slattery J, Murphy WJ, Antunes A, et al. (2006) The late Miocene radiation of modern Felidae: a genetic assessment. Science 311: 73-77.

S14. Kopefli KP, Deere KA, Slater GJ, Begg C, Begg K, et al. (2008) Multigene phylogeny of the Mustelidae: resolving relationships, tempo and biogeographic history of a mammalian adaptive radiation. BMC Biol 6: 10. doi:10.1186/1741-7007-6-10.

S15. Eizirik E, Murphy WJ, Koepfli KP, Johnson WE, Dragoo JW, et al. (2010) Pattern and timing of diversification of mammalian order Carnivora inferred from multiple nuclear gene sequences. Mol Phylogenet Evol 56: 49-63.

S16. Nyakatura K, Bininda-Emonds ORP (2012) Updating the evolutionary history of

Carnivora (Mammalia) a new species-level supertree complete with divergence time estimates. BMC Biol 10: 12. doi:10.1186/1741-7007-10-12.
S17. Mercer JM, Roth VL (2003) The effects of Cenozoic global change on squirrel phylogeny. Science 299: 1568-1572.
S18. Huchon D, Chevret P, Jordan U, Kilpatrick CW, Ranwez V, et al. (2007) Multiple molecular evidences for a living mammalian fossil. Proc N Acad Sci 104: 7495-7499.
S19. Steiner CC, Ryder OA (2011) Molecular phylogeny and evolution of the Perissodactyla. Zool J Linn Soc 163: 1289-1303.
S20. Revell LJ. 2010. Phylogenetic signal and linear regression on species data. Met Ecol Evol 1: 319-329.

APPENDICES

Appendix A. Limb inertial properties for hindlimbs (Table SA1) and forelimbs (Table SA2).

Table SA1. Hindlimb inertial propreties for each species included in this study.

Taxon	N	Body Mass	Limb Mass	Length	Center of Mass (COM)	Moment of Inertia (MOI)	Radius of Gyration	Natural Frequency
		(g)	(g)	(cm)	(cm)	($g*cm^2$)	(cm)	(Hz)
**Didelphimorphia**								
Didelphidae								
Didelphis virginiana	3	3240.0	163.7	23.6	7.5	15401.2	9.7	1.40
**Carnivora**								
Canidae								
Canis latrans	2	11488.0	1080.5	44.0	10.9	247425.6	15.3	1.08
Canis lupus	7	30772.9	2614.9	65.7	15.0	1141365.5	20.6	0.94
Lycaon pictus	1	22050.1	1650	63.6	19.5	1091081.1	25.7	0.86
Otocyon megalotis	1	3000.0	337.5	31.3	7.3	33940.1	10.0	1.34
Urocyon cinereoargenteus	5	3745.2	347.8	32.8	8.1	42719.2	11.2	1.27
Vulpes vulpes	1	3587.0	287	37.8	10.2	53074.6	13.6	1.17
Felidae								
Acinonyx jubatus	1	50000.0	3680	68.8	16.2	1928815.1	22.9	0.88
Caracal caracal	3	7050.0	1025.8	45.5	12.3	267769.7	16.1	1.09
Lynx rufus	4	8960.0	1066.2	42.3	10.7	231023.3	14.7	1.11
Puma concolor	1	68039.0	5900	71.1	21.2	4498940.8	27.6	0.83
Mephitidae								
Mephitis mephitis	1	2386.0	86	18.4	6.6	5035.3	7.7	1.68
Mustelidae								
Lontra canadensis	5	7287.2	323.2	24.4	6.9	25479.0	8.9	1.48
Martes pennanti	5	4661.6	393.6	29.3	8.5	51032.6	11.3	1.30
Mellivora capensis	2	15100.0	1032.5	32.4	10.2	177208.0	13.1	1.22
Mustela vison	1	886.0	59.9	13.7	5.0	2264.3	6.1	1.81
Taxidea taxus	4	6173.0	428	22.7	5.6	27272.6	8.0	1.48
Procyonidae								
Procyon lotor	6	6178.7	761.5	32.0	8.5	103930.7	11.5	1.27

**Artiodactyla**								
Bovidae								
Aepyceros melampus	2	51750.0	5750	84.1	14.0	2891139.0	21.9	0.85
Antidorcas marsupialis	3	39049.9	2625	67.3	8.5	428302.1	12.7	1.14
Connochaetes gnou	1	107450.0	9700	84.5	12.4	4625915.1	21.8	0.80
Kobus ellipsiprymnus	2	238000.0	23500	110.6	18.2	20210839.6	29.3	0.73
Oreotragus oreotragus	2	10500.0	1085	43.3	7.6	150943.5	11.8	1.18
Raphicerus melanotis	1	9750.0	1200	47.6	9.2	215220.5	13.4	1.13
Tragelaphus scriptus	1	54000.0	13000	69.1	9.0	3968938.9	17.5	0.86
Tragelaphus strepsiceros	2	236500.0	25000	131.1	21.2	29955607.1	34.5	0.67
Cervidae								
Cervus canadensis	5	241500.0	28200	122.3	23.1	38109032.6	36.7	0.65
Cervus elaphus	1	166562.5	5300	80.4	10.4	1711940.9	18.0	0.90
Odocoileus virginianus	4	69318.3	7631.3	91.9	20.8	6646695.7	29.5	0.77
Giraffidae								
Okapia johnstoni	1	230001.1	19400	113.7	27.0	27919838.3	37.9	0.68
**Cingulata**								
Dasypodidae								
Dasypus novemcinctus	2	4904.0	476.2	19.9	4.1	22512.7	6.9	1.46
**Hyracoidea**								
Procaviidae								
Procavia capensis	1	2250.0	140.0	18.9	4.3	5991.6	6.5	1.58
**Lagomorpha**								
Leporidae								
Sylvilagus floridanus	4	1130.5	120.3	24.4	6.1	8267.6	8.3	1.49

**Perissodactyla**								
Equidae								
Equus caballus	4	446250.3	43750	133.9	28.6	60962188.1	37.2	0.62
Equus quagga burchellii	2	266675.0	29875	107	15.1	28218199.8	30.6	0.64
**Rodentia**								
Castoridae								
Castor canadensis	5	16810.0	1213.6	31.2	8.9	183742.7	12.1	1.24
Cricetidae								
Ondatra zibethicus	4	1060.5	101.9	14.6	3.9	3003.1	5.4	1.82
Erethizontidae								
Erethizon dorsatum	1	5540.0	533	25.1	7.0	47468.9	9.43	1.40
Hystricidae								
Hystrix africaeaustralis	2	14936.0	1246	27.3	7.4	141128.3	10.4	1.31
Muridae								
Rattus rattus	4	330.9	28.4	9.8	2.8	403.5	3.7	2.21
Sciuridae								
Marmota monax	2	3542.0	278.0	19.4	4.9	11785.0	6.5	1.68
Sciurus carolinensis	4	527.8	61.8	14.2	3.6	1512.3	4.9	1.90
Sciurus niger	2	666.2	75.0	15.7	4.3	2476.0	5.8	1.79
Tamias striatus	4	97.4	8.8	7.2	1.8	51.5	2.4	2.79

TableSA2. Inertial propreties of the entire forelimb (i.e., inclusive of the scapula) for each species included in this study.

Taxon	N	Body Mass	Limb Mass	Length	Center of Mass (COM)	Moment of Inertia (MOI)	Radius of Gyration	Natural Frequency
		(g)	(g)	(cm)	(cm)	($g*cm^2$)	(cm)	(Hz)
**Didelphimorphia**								
Didelphidae								
Didelphis virginiana	3	3240.0	139.7	25.3	10.2	16840.6	10.9	1.48
**Carnivora**								
Canidae								
Canis latrans	2	11488.0	950.5	53.2	18.0	464894.6	22.2	0.95
Canis lupus	7	30772.9	2184.9	73.2	21.5	1631306.4	27.1	0.85
Lycaon pictus	1	22050.1	1500	69.6	27. 7	1575451.7	32.4	0.81
Otocyon megalotis	1	3000.0	218.5	32.3	9.0	29244.9	11.6	1.29
Urocyon cinereoargenteus	4	3745.2	222.4	33.0	10.5	40076.9	13.4	1.22
Vulpes vulpes	1	3587.0	225	38.0	12.4	50922.0	15.0	1.17
Felidae								
Acinonyx jubatus	1	50000.0	2456	70	14.6	1020368.5	20.4	0.93
Caracal caracal	3	7050.0	647.2	42.3	14.4	199279.7	17.5	1.09
Lynx rufus	5	8960.0	776.4	42.5	13.6	235980.0	17.2	1.07
Puma concolor	1	68039.0	4850	71.6	25.5	4787568.6	31.4	0.80
Mephitidae								
Mephitis mephitis	1	2386.0	79	19.4	8.2	5646.1	8.5	1.69
Mustelidae								
Lontra canadensis	5	7287.2	291.4	23.5	7.9	24644.0	9.2	1.53
Martes pennanti	5	4661.6	319.8	29.2	10.0	48954.9	12.1	1.30
Mellivora capensis	2	15100.0	1165	39.9	14.3	329191.8	16.8	1.12
Mustela vison	1	886.0	48.1	14.0	5.7	2018.4	6.5	1.83
Taxidea taxus	4	6173.0	441.5	29.7	10.0	66235.7	12.1	1.30
Procyonidae								
Procyon lotor	6	6178.7	411.9	32.4	10.1	66715.1	12.7	1.27

**Artiodactyla**								
Bovidae								
Aepyceros melampus	2	51750.0	2875	90.0	25.9	3058626.6	32.8	0.77
Antidorcas marsupialis	3	39049.9	1255	68.3	17.5	582121.5	21.5	0.97
Connochaetes gnou	1	10745.0	5900	98.0	32.0	9034775.4	39.1	0.72
Kobus ellipsiprymnus	2	238000.0	12875	120.0	39.6	30174250.1	48.4	0.65
Oreotragus oreotragus	2	10500.0	481.5	45.2	13.1	125156.3	16.1	1.12
Raphicerus melanotis	1	9750.0	465	47.6	12.9	116862.5	15.9	1.13
Tragelaphus scriptus	1	54000.0	2950	80	25.0	2680345.5	30.1	0.83
Tragelaphus strepsiceros	2	236500.0	15250	144.1	45.4	49787285.4	57.17	0.59
Cervidae								
Cervus canadensis	5	241500.0	16050	137.3	40.7	44632148.1	52.7	0.60
Cervus elaphus	1	166562.5	2500	82	24.8	2312308.1	30.4	0.82
Odocoileus virginianus	4	69318.3	3693.8	98.1	32.6	5851739.9	39.7	0.72
Giraffidae								
Okapia johnstoni	1	230001.1	17700	149.1	54.2	77473424.9	66.2	0.56
**Cingulata**								
Dasypodidae								
Dasypus novemcinctus	2	4904.0	179.7	18.7	7.1	11497.6	8.0	1.67
**Hyracoidea**								
Procaviidae								
Procavia capensis	1	2250.0	104.0	19.6	7.0	7190.4	8.3	1.59
**Lagomorpha**								
Leporidae								
Sylvilagus floridanus	3	1130.5	56.3	20.9	7.3	4234.9	8.6	1.56

**Perissodactyla**								
Equidae								
Equus caballus	4	446250.3	32750.0	134.0	37.3	86253441.7	51.3	0.59
Equus quagga burchellii	2	266675.0	15850.0	116.9	35.5	31566975.7	44.8	0.66
**Rodentia**								
Castoridae								
Castor canadensis	5	16810.0	460.8	26.18	10.0	65652.1	11.7	1.36
Cricetidae								
Ondatra zibethicus	4	1060.5	47.6	12.4	5.0	1531.5	5.6	1.99
Erethizontidae								
Erethizon dorsatum	1	5540.0	328	25.1	9.2	37842.7	10.7	1.41
Hystricidae								
Hystrix africaeaustralis	2	14936.0	980.5	31.9	11.9	202003.5	14.1	1.22
Muridae								
Rattus rattus	4	330.9	15.3	8.6	3.2	207.7	3.7	2.46
Sciuridae								
Marmota monax	2	3542.0	219.0	20.4	6.4	12611.6	7.6	1.66
Sciurus carolinensis	4	527.8	35.1	13.0	4.6	1001.3	5.3	2.00
Sciurus niger	2	666.2	44.4	14.3	5.1	1609.8	5.9	1.89
Tamias striatus	4	97.4	5.4	6.6	1.9	26.8	2.2	3.13
